# Multi-country metabolic signature discovery for chicken health classification

**DOI:** 10.1007/s11306-023-01973-4

**Published:** 2023-02-02

**Authors:** Joanna C. Wolthuis, Stefanía Magnúsdóttir, Edwin Stigter, Yuen Fung Tang, Judith Jans, Myrthe Gilbert, Bart van der Hee, Pim Langhout, Walter Gerrits, Arie Kies, Jeroen de Ridder, Saskia van Mil

**Affiliations:** 1 Center for Molecular Medicine, University Medical Center Utrecht and Utrecht University, STR3.217, PO Box 85060, 3508 AB Utrecht, The Netherlands; 2 Oncode Institute, Utrecht, The Netherlands; 3 Animal Nutrition Group, Department of Animal Sciences, Wageningen University and Research, Wageningen, The Netherlands; 4 Host-Microbe Interactomics, Department of Animal Sciences, Wageningen University and Research, Wageningen, The Netherlands; 5 DSM Nutritional Products, Animal Nutrition and Health, Kaiseraugst, Switzerland; 6 Department of Environmental Microbiology, Helmholtz Centre for Environmental Research-UFZ, Leipzig, Germany

**Keywords:** Mass spectrometry, Machine learning, Gut, Inflammation, Enrichment, Chicken

## Abstract

**Introduction:**

To decrease antibiotic resistance, their use as growth promoters in the agricultural sector has been largely abandoned. This may lead to decreased health due to infectious disease or microbiome changes leading to gut inflammation.

**Objectives:**

We aimed to generate a m/z signature classifying chicken health in blood, and obtain biological insights from the resulting m/z signature.

**Methods:**

We used direct infusion mass-spectrometry to determine a machine-learned metabolomics signature that classifies chicken health from a blood sample. We then challenged the resulting models by investigating the classification capability of the signature on novel data obtained at poultry houses in previously unseen countries using a Leave-One-Country-Out (LOCO) cross-validation strategy. Additionally, we optimised the number of mass/charge (m/z) values required to maximise the classification capability of Random Forest models, by developing a novel ranking system based on combined univariate t-test and fold-change analyses and building models based on this ranking through forward and reverse feature selection.

**Results:**

The multi-country and LOCO models could classify chicken health. Both resulting 25-m/z and 3784-m/z signatures reliably classified chicken health in multiple countries. Through *mummichog* enrichment analysis on the large m/z signature, we found changes in amino acid metabolism, including branched chain amino acids and polyamines.

**Conclusion:**

We reliably classified chicken health from blood, independent of genetic-, farm-, feed- and country-specific confounding factors. The 25-m/z signature can be used to aid development of a per-metabolite panel. The extended 3784-m/z version can be used to gain a deeper understanding of the metabolic causes and consequences of low chicken health. Together, they may facilitate future treatment, prevention and intervention.

**Supplementary Information:**

The online version contains supplementary material available at 10.1007/s11306-023-01973-4.

## Introduction

Due to the rise of antibiotic resistance and a growing public awareness of health and food safety issues, the chronic use of antibiotics as growth promotors in chicken has been forbidden in many countries (Roth et al., 2019). As a result, however, this may lead to lower performance, higher mortality, and deteriorated animal welfare. Such suboptimal health conditions are often characterised by a high incidence of wet litter (WL), which may cause footpad dermatitis and hock burns (Bessei, 2006). This encouraged researchers to investigate the origin of WL and low chicken health in order to combat the problem through approaches that do not require chronic antibiotic use. As reviewed by Gilbert et al. (2018), gut health aetiology is multifactorial and has been connected to both parasites and bacterial pathogens in the intestinal tract.

However, due to differences in housing environment, management practices and genetic strain of chickens used, the physiological causes may differ between countries and farms, making health classification a challenge. Gaining more insight into the molecular signature of chicken health is highly desired to facilitate future treatment, prevention and intervention. The current state-of-the-art metabolomics technology allows for the generation of decision-making tools that enable data-driven health monitoring, enabling timely interventions to improve animal health.

Direct infusion mass spectrometry (DI-MS) is an untargeted metabolomics method which only requires a single drop of blood, is fast, scalable to high-throughput and involves no chromatography. DI-MS has the additional advantage that no pre-selection is done and thus a global overview of the entire metabolome is collected in a matter of minutes (de Sain-van der Velden et al., 2017). Annotation of identified m/z values in DI-MS is the largest challenge, as it is done on highly accurate m/z values with no additional information, unlike in other mass spectrometry approaches where the retention time is used in addition to m/z to annotate the metabolites (D’Atri et al., 2017). To streamline statistical analysis and annotation, we developed a software solution, *MetaboShiny* and its companion database suite *MetaDBparse* (Wolthuis et al., 2020).

A metabolite signature should be independent of the genetic background, farm conditions, country of origin and food source because of the potential different aetiologies of chicken health across countries. This is necessary to interpret the biological insights from the resulting signature on a global level. Metabolites defining such a signature provide valuable information on the biological processes underlying chicken health, forming a foundation on which to build preventative solutions and interventions.

In this manuscript, we collected blood samples of both healthy and unhealthy chickens from three countries from two continents, from 22 farms in total, and used machine learning methods to define two metabolic signatures characteristic for chicken health.

## Methods

### Sample collection

Samples were obtained from 197 individual broiler chickens from 22 farms spanning 3 countries. Per chicken, four drops of blood were sampled in independent spots on “Whatman^tm^ 903 Protein Saver Cards” blood spot cards (BSC) (Sigma-Aldrich Merck KGaA, Darmstadt, Germany). Countries were selected based on availability of on-site experts to perform the sampling and willingness of local farmers to participate in the experiment. Furthermore, the participating countries are important chicken-producing countries in their respective continents and thus well suited for an initial impression of the variation present in chicken metabolomes. In general, each continent uses a different basal feed composition based on agricultural availability, which was an additional reason for including multiple countries across continents.

Sampling was performed by the country broiler expert/coordinator together with the local farmer. Prior to sampling, instructions and BSCs, together with sealing bags, moisture indicators and desiccant bags were sent by the UMC Utrecht team to the country expert/coordinator. The instruction video provided to the on-site experts for sampling can be found at https://tinyurl.com/bscumc.

**Fig. 1:**
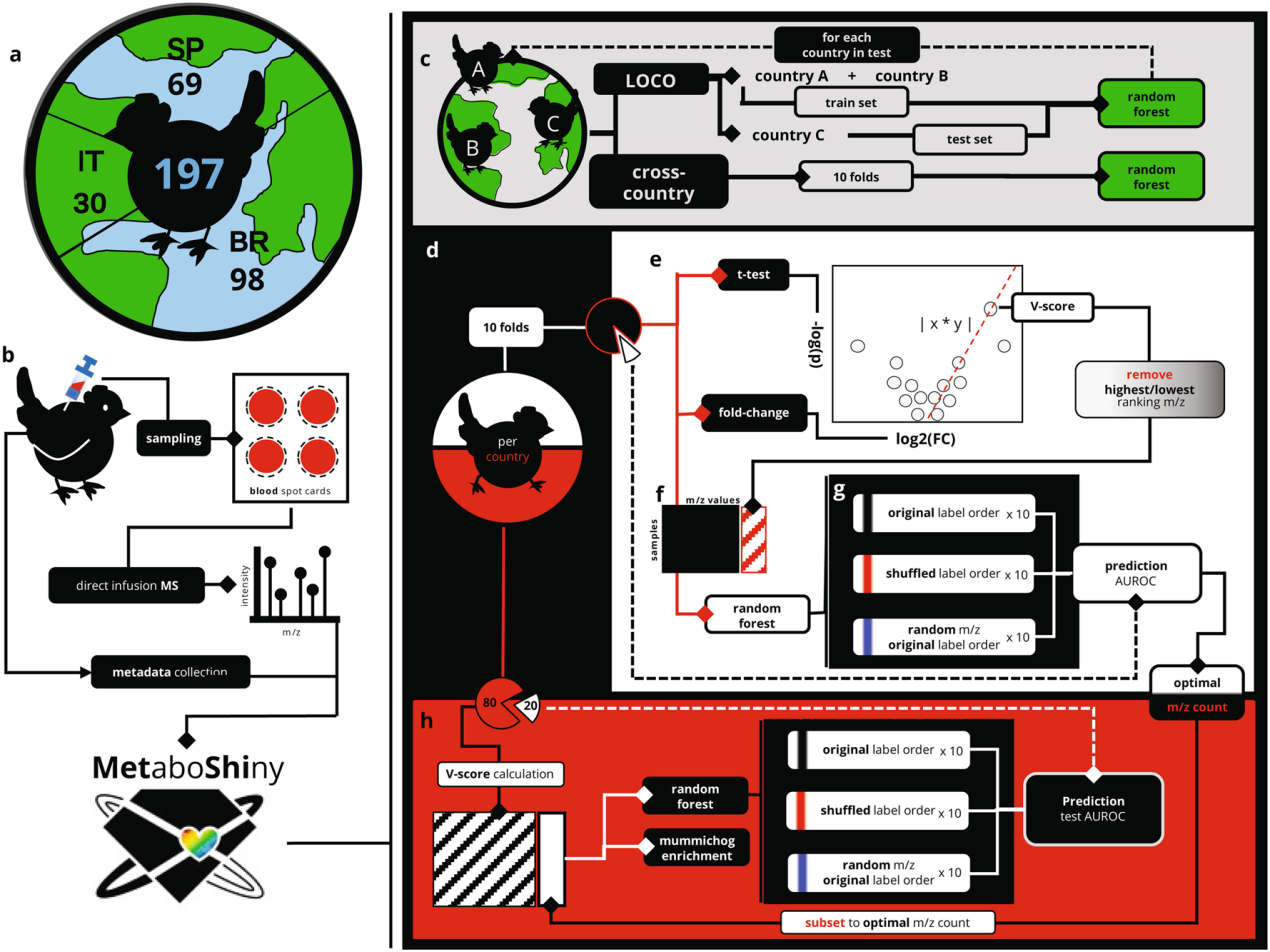
Using multi-country metabolomics data from blood drawn from chickens to find a molecular signature for chicken health. **a**, **b** Sample collection, mass spectrometry and pre-processing through *MetaboShiny*. SP = Spain, IT = Italy, BR = Brazil. **c** ‘Leave-One-Country-Out’ analysis used one left out country as the testing set in a random forest (RF) model, as opposed to a regular train/test split. This process was repeated for each country. **d** 50% of samples of each country were used for either feature selection or machine learning. **e** For feature selection, t-test and fold-change analyses were combined into a volcano plot and the ‘V-score’ (see Methods) was calculated per m/z. **f** The top or bottom ranking m/z values were used to limit the number of m/z values used in the subsequent feature selection step. **g** For each m/z selection, 3 types of models were built. Original label order representing the v-score, non-permuted data, shuffled labels representing negative control on the class label, and an equal number of random m/z values and regular label order. **h** With the optimal number of features, the final model was built and evaluated on a separate test set

In this rather uncontrolled field experiment, we wanted to capture a maximum amount of variation, to understand whether metabolic health classification is feasible on the basis of the perceived overall health status of the broilers as classified by the local farmers and country experts. Rather than registering specific characteristics, country experts registered a healthy (‘high’ health) and unhealthy (‘low’ health) label for each chicken, on the basis of several commonly seen and widely accepted characteristics: reduced animal size relative to the farm average, rough feathers, reduced activity, and/or presence of dirty feathers near the vent (Fig. 1a).

Each farm sampled 50% healthy and 50% unhealthy chickens regardless of on-site health distribution. Blood was drawn through syringe from the brachial wing vein and dripped onto the BSC. In total, Brazil supplied 98 BSCs distributed across 10 farms, Italy supplied 30 BSCs distributed across 5 farms and Spain supplied 69 BSCs distributed across 7 farms.

After being dried at room temperature, ranging from 5 h to overnight, the BSCs were stored in sealed plastic bags with desiccant and moisture level monitoring cards and stored at 4 °C. After collection of cards from all farms within a country was completed, they were shipped to the UMC Utrecht (transit time: 2–7 days) for further processing, Upon arrival BSCs were stored at -80 °C until analysis. BSCs on which the blood spots were not dripped as instructed or those that contained smears were excluded from the analysis.

### Data acquisition

Data acquisition was performed using our in-house pipeline for direct infusion mass spectrometry, as described by (de Sain-van der Velden et al., 2017). In short, from each blood spot card a 3 mm disk was punched and extracted in an ultrasonic bath with acetonitrile, formic acid and internal standards. Run order was randomised based on farm and, if multiple countries were measured simultaneously, country.

Following extraction, the samples were filtered and subjected to chip-based nano electrospray DI-MS analysis in positive and negative mode using an Advion TriVersa Nanomate combined with a Thermo Scientific Q-Exactive HF high resolution mass spectrometer. In positive and negative ion modes, the signal was collected for 3 min and 1.5 min, respectively, using a Thermo Scientific Q Exactive high resolution mass spectrometer. Three technical replicates were measured per blood spot and the resulting signal was saved as a.raw Thermo file for each replicate. The raw files and metadata including country, breed, temperature at sampling, sex, and feed type is hosted on *MetaboLights* alongside the raw data.

### Processing

*ThermoFileReader* was used to convert raw files to.*mzML*. Using the *xcms* package, files were divided into positive and negative mode scans based on the available metadata (Domingo-Almenara & Siuzdak, 2020). Samples with a low total intensity in either positive or negative mode were excluded from further analysis.

Scans were aligned to each other per mode and replication, using a list of m/z values observed in > 80% of samples, only found in internal standards. The spectra of each mode were then collected, yielding one positive and one negative spectrum per sample. Per sample, the *MALDIquant* program was used to call peaks (Gibb & Strimmer, 2012). Next, all files associated with the aforementioned experiments were gathered, and a summary of all peak tables was created. To facilitate statistical analysis, spectra were binned/aligned to each other using *MALDIquant* at 2 ppm (parts per million).

The binned spectra were combined into a table and exported to a *MetaboShiny*-supported table. Only the peaks observed in 2/3 technical triplicates were kept, and the peak signals of the replicates were averaged. A table including data on the day-of-run batch and injection order was also exported for batch correction purposes. The raw file pipeline can be found on Github for SLURM-supporting compute clusters in the *joannawolthuis/MassChecker* repository.

### Filtering and normalization

Peaks with more than 20% missing samples were excluded from the analysis based on Bijlsma et al. (2006)’s recommendation. Peak intensities were quantile-adjusted for each sample and then auto-scaled to Z-score format per m/z value. The *WaveICA* package was then used to correct any batch effect, using both the day-of-run batch and injection order (Chong & Xia, 2018; Deng et al., 2019; Gibb & Strimmer, 2012; Wolthuis et al., 2020). Post batch-correction, batches no longer clustered together majorly in UMAP (Fig. S1).

### Machine learning and cross-validation

We used the Random Forest (RF) model as implemented in the *caret* package for training predictive models. For the Leave-One-Country-Out (LOCO) experiment, we built a model for each country, with the *mtry* parameter of the model set to the square root of all m/z values available in the dataset. The LOCOCV Receiver Operator Characteristic (ROC) and Precision-Recall (PR) curves were calculated by combining the country-fold classifications.

Before any analysis takes place the dataset is split in two equal folds using *caret*’s *createFolds* function (Fig. 1a). One fold (50% of the data) is used for feature selection and determination of the number of top ranking m/z values. Within the other 50% data fraction, 80% of the data was used to construct a model on the top N m/z values ranked on the V-score, where N is determined by the feature selection procedure (see below). The resulting RF model was tested on the remaining 20%, and the model creation and testing process was repeated 10 times.

### Feature selection

Within the feature selection fold, we further defined 10 folds. In each fold, we performed t-test and fold-change analyses through *MetaboShiny*. To enable ranking of m/z-values in further steps we use the T-test -log(p) and log2(FC) outcome of the fold-change analysis. To this end, we visualised these two outcomes in a volcano plot and calculated a combined V-score per m/z value.

$$ {\text{V - score}} = - \log_{10} \left( {\text{p - value}} \right) \times \log_{2} \left( {\text{fold - chang}} \right) $$

The columns in the dataset were ranked in order of descending absolute V-score (Fig. 1b). We next evaluated classification capability for subsets of the datasets by progressively adding or removing m/z columns according to the V-score ranking. For each evaluation the *mtry* parameter of the RF model was set to the square root of the amount of m/z values present in that subset of the dataset (Fig. 1c).

For each evaluation we included two negative controls. Negative control 1 (‘*shuffled’*) is defined as the dataset using permuted class labels, which acts as a control of the classification quality. Negative control 2 ('*randomised m/z’*) is defined as the dataset using a randomly selected same-sized set of m/z values taken from the complete dataset, which was used to assess if the V-score ranked classifier outperforms a classifier based on randomly selected m/z columns. The final number of selected m/z values was determined by comparing the AUROC for negative control 2 to the V-score ranked dataset (Fig. 1d).

To obtain a compact signature, models were first built adding 10 m/z values per step (starting with 2, 12, 22, etc.), and additionally on a per-m/z basis for the top 300 m/z values to more accurately determine the optimum amount of m/z values necessary.

For the experiment removing high-ranking m/z values (‘expanded’ signature), the complete experiment used models built by removing 10 m/z values per step. For each subset of m/z values, the tenfold cross-validated AUROC was calculated. Lines were fit to the V-score ranked and both negative control AUROCs using *ggplot2*’s *geom_smooth* function.

For both signature determinations, the line fit summarising the V-score-ranked AUROC was used. For the compact signature, peak detection was used to find a peak in the top 300 V-score-ranked m/z values. For the expanded signature, the elbow point of the line removing m/z values in descending absolute V-score order (high-ranking first) was used as the signature threshold. The m/z values included in the expanded signature were used in the enrichment analysis.

### Correlation analysis

Correlation between m/z values was calculated using the *cor* function in R, specifying Pearson correlation. We visualised the heatmap using the *ggplot2* package, where only correlation pairs with a p-value < 0.05 were coloured.

### Enrichment

Using the expanded signature, we performed *mummichog* enrichment analysis using the *MetaboShiny-*integrated *MetaboAnalystR* package. We adapted the algorithm to use adducts selected by the user (in our case the adducts provided in Table S1). Necessary pathway databases ‘*Gallus gallus’* (KEGG ID: *gga01100*) and ‘*microbial metabolism in diverse environments’ (KEGG ID: map01120)* were newly generated using the *build.pathway.KEGG* function which uses the *KEGGREST* package to retrieve necessary organism, pathway and compound information. In addition, custom adducts were generated for each compound (table S1).

The KEGG chicken pathway collection includes amino acid synthesis pathways for amino acids that chickens cannot synthesise. For this reason, after first downloading the complete *gga01100* pathway collection, we next removed pathways involved in synthesising any of the thirteen chicken essential amino acids: arginine, cysteine, histidine, isoleucine, leucine, lysine, methionine, phenylalanine, proline, threonine, tryptophan, tyrosine and valine as described by He et al. (2021). To that end, pathways featuring the following modules were excluded: ‘*proline biosynthesis, glutamate* > *proline’*, ‘*cysteine biosynthesis, homocysteine* + *serine* > *cysteine*’, and *‘arginine biosynthesis, ornithine* > *arginine*’. These filtering steps lead to the exclusion of the pathways ‘*arginine biosynthesis’*, ‘*valine, leucine and isoleucine biosynthesis’* and ‘*phenylalanine, tyrosine and tryptophan biosynthesis’.*

In determining the enriched pathways in the expanded signature, we relied on the previously published *mummichog* method (Li et al., 2013)*.* A predetermined amount of m/z values was marked as significant based on if they were present in the 3784-m/z signature or not. Enriched pathways with *mummichog* EASE p ≤ 0.05 were included in further interpretation.

### Visualization

We used the *ggplot2, ggrepel*, *ggbeeswarm, ggsignif, ggforce, moonBook, visNetwork* and *plotly* packages, most through our application *MetaboShiny,* to visualise most results (Sievert et al., 2016; Wickham, 2011). An adapted version of the *pathview* package was implemented in *MetaboShiny* and used to generate KEGG metabolic pathway images and project V-scores onto these pathway diagrams (Luo & Brouwer, 2013).

## Results

### Leave-One-Country-Out analysis demonstrates model flexibility

Our goal was to define a chicken health m/z signature that is globally applicable and thus may also be applied to chicken samples from other countries not presented in the training dataset, irrespective of environment, housing and feeding conditions. To emulate this scenario, we designed and applied a ‘Leave-One-Country-Out’ (LOCO) strategy, where data from one country were excluded from the training set, and used as the testing set instead.

**Fig. 2:**
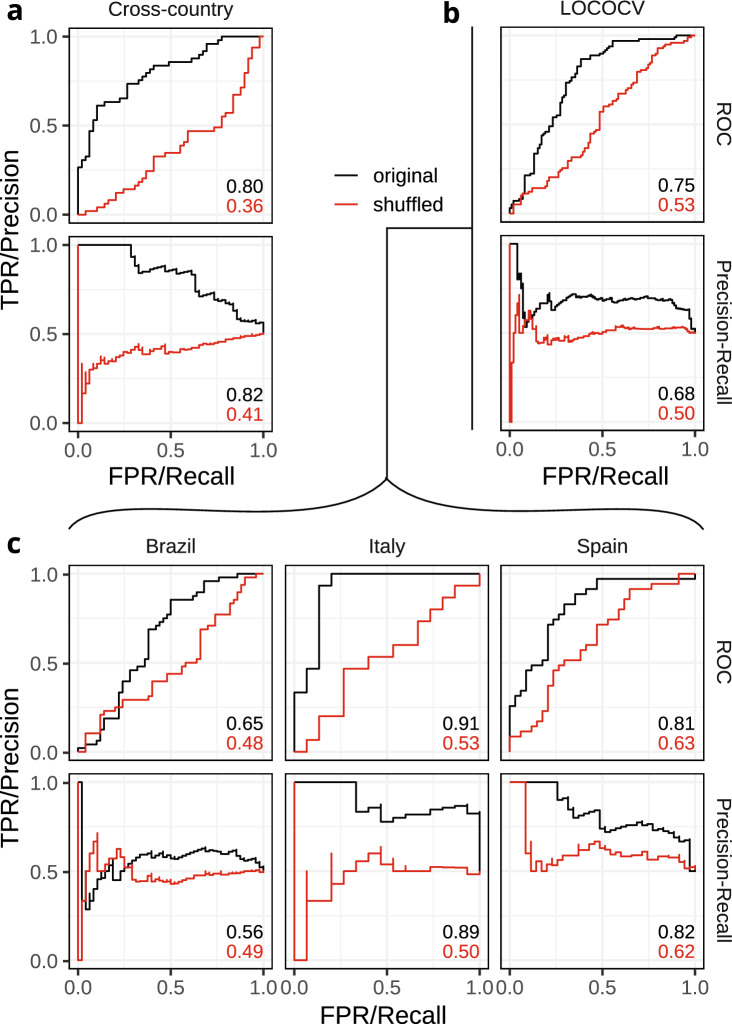
AUROC and AUPRC of LOCOCV models. AUC of both original and shuffled models is displayed in the lower right of each curve. **a** LOCOCV curves were drawn and evaluated by combining the three separate ‘folds’ of LOCO analysis into one pool and calculating the resulting complete cross-validated curves. **b** Per-country LOCO model evaluation. Country in header indicates country used as testing set. **c** ‘Multi-country’ model is a general model evaluated using tenfold CV without removal of specific countries

We first used Random Forest (RF) classifiers to construct classifying models whilst allowing the RF access to all m/z features, i.e. no feature selection was part of this setup (Fig. 1c). Figure 2 reports the AUROC (Area Under the Receiver Operating Characteristic) and AUPRC (Area Under the Precision-Recall Curve) for each model.

We built two types of models; a multi-country model and a Leave-One-Country-Out model. For the multi-country model tenfold cross validation was used, where the splits were stratified based on health label and country. This model used samples from all countries in both the training and testing set. The model reached an AUROC of 0.80 and AUPRC of 0.82 (Fig. 2a). As a negative control, we include a model using permuted health labels and these models showed low classification capability as the AUROC and AUPRC were close to 0.5.

To train the LOCOCV model, we used threefold cross validation (CV) such that each left out fold was a complete country (Fig. 2b). The LOCOCV model is aimed at estimating how well the model would do on a novel country, where the desired maximum AUROC/AUPRC would be close to the multi-country model. The resulting model had an AUROC of 0.75 and AUPRC of 0.68.

The LOCOCV model has a similar classification capability to the multi-country model (ΔAUROC = 0.05), and this suggests both inter-country similarity and potential usability of the multi-country model in novel countries.

To give further insight into which countries had the most classification capability, we dissected the LOCOCV curve into its separate test countries (Fig. 2c).

The LOCO model tested on Italy and trained on the other countries classified best with an AUROC of 0.91 and AUPRC of 0.89. The Spain models had an AUROC of 0.81 and AUPRC of 0.82, and Brazil had an AUROC of 0.65 and AUPRC of 0.56.

In two out of three country folds, the model classifies better than the multi-country model, further suggesting inter-country similarity in terms of health metabolomic signature and suitability of the multi-country model for use in novel countries.

### Compact 25-m/z signature stratifies health with high accuracy

Next, we aimed to create a model that is both compact and optimally discriminative, as a model with few metabolites facilitates the creation of a testing panel in future applications, where metabolite concentrations can be quantified on an a per-metabolite basis using preselected standards. To find the minimum set of metabolites required for accurate classification, we performed a forward feature selection strategy. To this end, we first ranked the m/z values based on the ‘V-score’, a score combining T-test and fold-change analysis results.

**Fig. 3:**
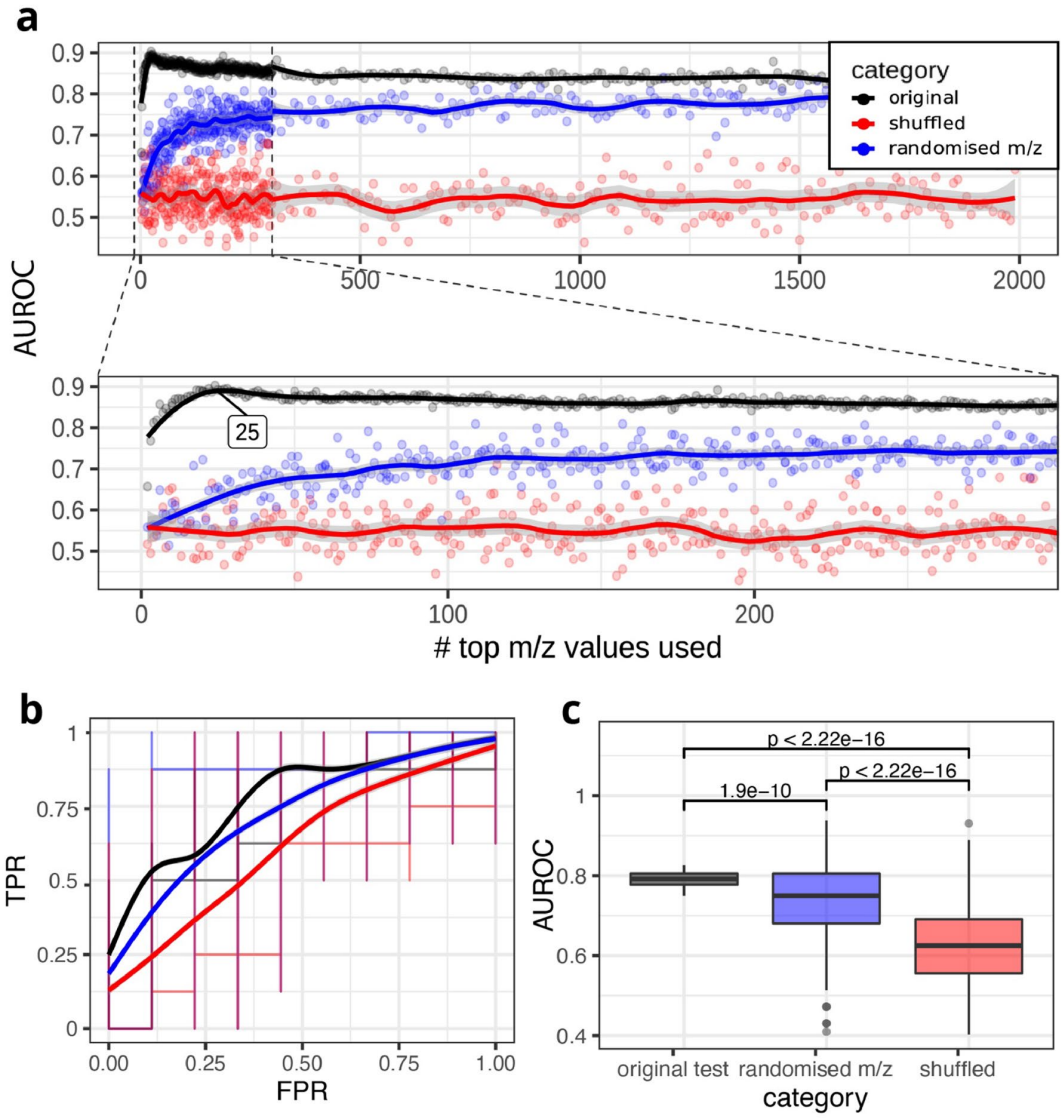
Using V-score ranking to find the smallest most classifying set of top ranking m/z values. **a** AUROC plot against the number of m/z values used. Bottom panel zooms in on the top m/z values. Calculated peak of the fit curve is labelled at x = 25. The grey shading around each fit visualises the 95% confidence interval of the line fit by the geom_smooth algorithm. **b** AUCs of 10 original ranked models vs. 10 randomised m/z models. T-test p-value is noted above the boxplots. **c** ROCs of test sets of 10 original models, 10 shuffled models using original m/z order, and 10 models using randomised m/z values

To estimate the optimal number of m/z values that should be used in a model, we compared the AUROC of models using m/z values ranked by decreasing absolute V-score (black curve) with the AUROC of a model using a random equal number of m/z values as a negative control (blue curve) for all top ranking m/z values, starting with top 2 up to 7672 m/z values (Fig. 3a). We observed the highest AUC for a model trained on 25 m/z values, referred to as the compact signature. Importantly, at this threshold, the model classified better than a model using 25 random m/z values. Furthermore, using fewer m/z values caused a rapid decrease in AUROC.

In the validation set, the average AUROC of the compact signature was above both the random 25-m/z model and shuffled health label models (Fig. 3b), demonstrating that a model based on the top 25 m/z values was significantly more effective (*p* = *1.9e-10*; t-test) than using randomly selected m/z values in an independent validation set.

Furthermore, as shown in Fig. 3c, both using the compact signature and a random signature classify significantly better than a negative control model with permuted health labels (signature vs. shuffled*: p* < *2.2e−16*, randomised vs. shuffled: *p* < *2.2e−16, t-test*).

### Model interpretation using mummichog enrichment analysis

**Fig. 4:**
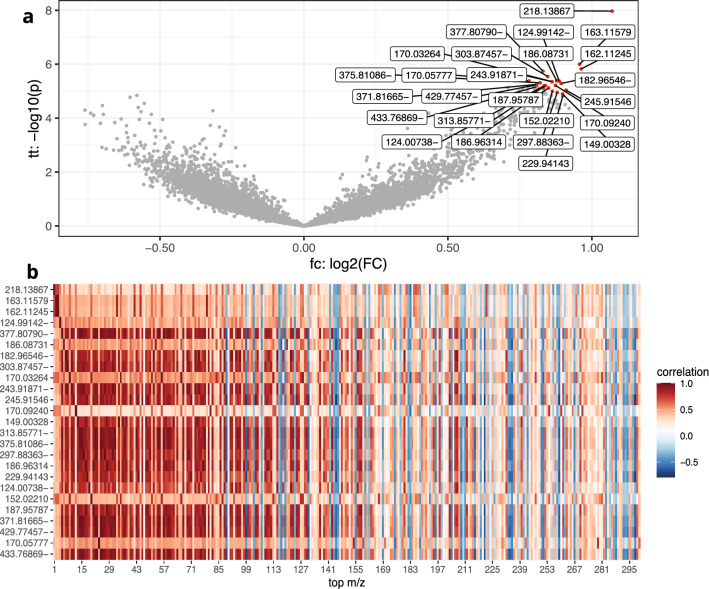
M/z values are highly correlated. **a** Volcano plot of the 25-m/z signature. **b** Heatmap of Pearson correlation between the top 25 (y-axis) and top 300 V-score ranked (x-axis) m/z values

To gain biological insights and understand which metabolic pathways play a role in chicken health, we performed enrichment analysis of the metabolites in the signature. However, while the compact signature worked well for classifying chicken health, due to the presence of adducts and metabolic pathway interactions, the metabolites corresponding to the 25 m/z-values likely do not capture the full biological signal (Fig. 4a).

To quantify the degree of correlation in the data, we calculated the Pearson correlation between each pair of V-score ranked top 300 m/z values. The m/z values in the top 25 signature were often highly correlated to m/z values within the top 300 (Fig. 4b). Therefore, we defined a second signature capturing the full biological signal including all correlated m/z. This signature used more m/z values than strictly necessary for an accurate classification, but included the complete set of m/z values defining what differentiated healthy and unhealthy chickens and may therefore be more suitable for interpretation.

**Fig. 5:**
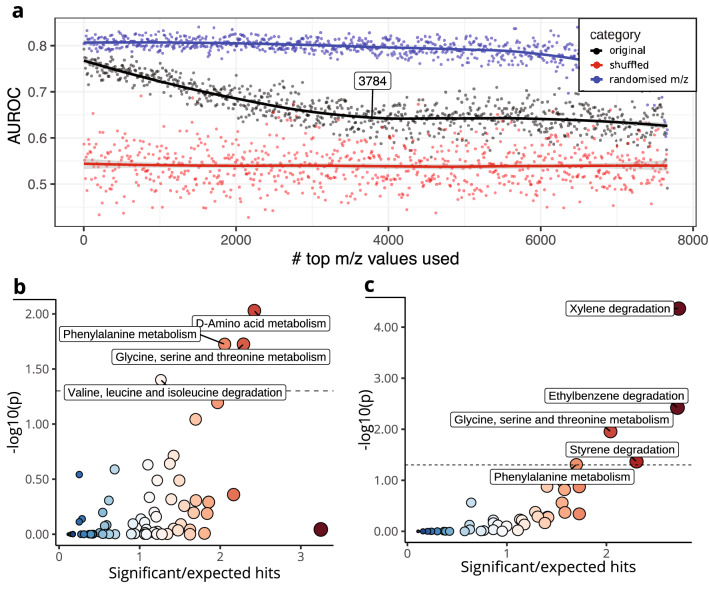
Determining expanded V-score based m/z signature. **a** Black line represents non randomised health based on V-score-ordered m/z values; peaks in this line are of interest. Blue line represents models made with random m/z values. Red line represents label-shuffled models. The grey shading around each fit visualises the 95% confidence interval of the line fit by the *geom*_*smooth* algorithm. **b** Enrichment analysis result using the KEGG *gallus gallus* pathway collection. **c** Enrichment results using the pathways in the KEGG *microbial metabolism in diverse environments* pathway collection

The expanded signature size was defined as the number of features that needed to be removed before the difference between a completely random classifying model and our model missing top ranking features reached a low plateau. This resulted in selection of the 3784 top ranking m/z values in our expanded signature (Fig. 5a).

Pathway enrichment analysis was performed using the m/z values in the expanded signature. Only a limited number of methods is available to achieve this for untargeted metabolomics data. We utilised the *mummichog* enrichment method within the *MetaboAnalystR* package and adapted for custom adducts and pathway filtering, integrated in *MetaboShiny* (Chong et al., 2018; Li et al., 2013; Wolthuis et al., 2020). We first ran *mummichog* using the *gallus gallus* KEGG pathway collection (Fig. 5b, Table S2).

In this pathway collection*,* four pathways were significantly altered: *‘D-amino acid metabolism’, ‘glycine, serine and threonine metabolism, ‘phenylalanine metabolism’* and ‘*valine, leucine and isoleucine degradation*’. Furthermore, we included potential bacterial metabolites being produced and absorbed into the bloodstream, such as bacterial protein fermentation end products known to impact host metabolism (Gilbert et al., 2018). We did so by searching the ‘*microbial metabolism in diverse environments’* KEGG pathway collection. Two pathways were significantly enriched, ‘*glycine, serine and threonine metabolism’ and ‘phenylalanine metabolism’*, both of which were also significantly enriched in the *gallus gallus* pathway collection (Fig. 5c, Table S3).

**Fig. 6:**
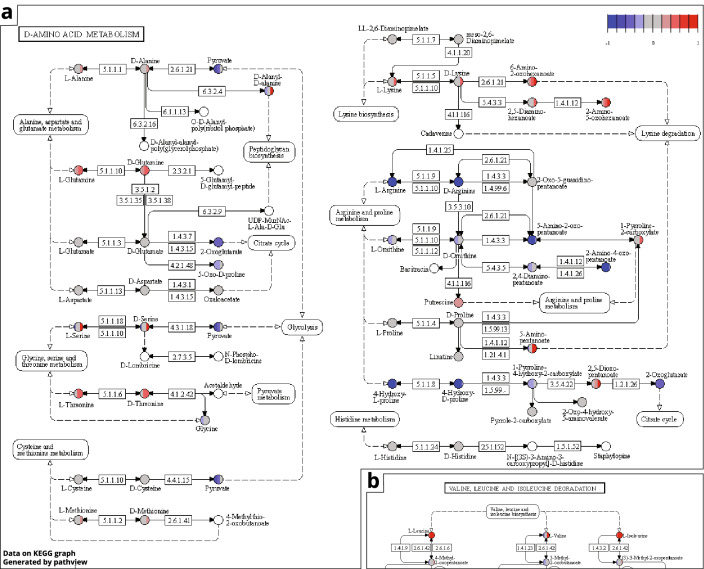
**a** Visualisation of the ‘D-amino acid metabolism’ KEGG pathway, the largest affected pathway featuring most discussed amino acids. **b** Part of the *‘*valine, leucine and isoleucine degradation’ KEGG pathway. Colour of nodes indicates V-score. Vertical lines within a node represent V-scores for multiple adducts of a given compound. Grey nodes represent metabolites with potential matches outside of the signature used for enrichment. Pathway image was generated using the R *pathview* package

We projected the V-score onto the top ranking enriched pathways (Fig. 6, Figs. S2–S4) and compared our results to previously conducted pathway enrichment experiments across species and in poultry, relying on the fact that *mummichog* compound matches are more likely to be true matches due to random matches not being enriched within pathways (Li et al., 2013). With that in mind, this allowed for a careful interpretation of individual metabolites in the context of poultry health, gut health, feed intake and body weight gain, while it should be noted that these are putative annotations, annotated at level 2 (Sumner et al., 2007).

In *D-amino acid metabolism* we saw potential increases in unhealthy chickens in lysine, diamino-hexanoate, amino-oxohexanoate, glutamine, threonine, cysteine, methionine, putrescine, pyrrole-carboxylate, dioxopentanoate, phenylalanine and serine, and potential decreases in alanine, pyruvate, arginine, hydroxyproline, oxoglutarate, and amino-oxopentanoate (Fig. 6a, Table S4). Within the *valine, leucine and isoleucine degradation* pathway, we saw potential increases in leucine and valine, alongside metabolites hydroxyisovalerate and hydroxyisobutyrate, alongside decreases in aminoisobutanoate, methyl-oxobutanoate and aminoisobutanoate (Fig. 6b, Fig. S2, Table S5). *Glycine, serine and threonine metabolism* pathway members affected were mainly ectoine-related compounds such as ectoine, 5-hydroxy-ectoine and n-acetyl-2,4-diaminobutyrate, which were increased in the unhealthy group, alongside creatine, cysteine serine, threonine, homoserine, propane-1,3,-diamine, allothreonine, glycerate and phospho-d-glycerate. Furthermore, we observed potential decreases in pyruvate, 5-aminolevuliate, 5-aminolevulinate, O-phospho-homoserine and dimethylglycine (Fig. S3, Table S6). Within *phenylalanine metabolism* we again mainly saw decreases in the unhealthy group in tyrosine, phenylethylamine, 2,6-dihydroxyphenylacetate, phenylacetamide, phenylpyruvate, N-Acetyl-phenylalanine, hydroxyphenylpropanoate, phenylacetaldehyde, phenylethylalcohol, 2-hydroxy-3-phenylpropanoate, 2-hydroxy-6-oxonona-2,4-diene-1,9-dioate, cis-2-hydroxypenta-2,4-dienoate and, again, pyruvate. 4-Hydroxy-2-oxopentanoate was however increased in the unhealthy group, alongside possibly phenylalanine, phenylacetate, phenylglyoxylate and phenylpropanoate (Fig. S4, Table S7).

These results demonstrated that the health signature was enriched in changes to m/z values corresponding to metabolites featured mainly in amino acid metabolic pathways. This suggests that the healthy and unhealthy chickens differ in amino acid metabolism.

## Discussion and conclusion

To build models classifying chicken health and use these models to gain insight in the potential causes and metabolic consequences, we investigated the connection between the chicken blood metabolome and health label. We explored this connection in order to test if metabolic differences exist between the two groups, and if so, gain biological insights from the resulting m/z signatures.

We first aimed to gain knowledge that is not specific to a single country which is why we collected samples from three countries on two continents. However, to determine if the multi-country model could also classify chicken health in a country not presented in the training dataset, we designed the ‘Leave-One-Country-Out’ (LOCO) experiment. This was with the assumption that intra-country and farm-based differences would likely be smaller than inter-country differences in living conditions and feed. Overall, the similar classification success of the LOCOCV and multi-country model, alongside the AUCs of on average 0.8 of the separate folds of LOCOCV, suggests that the multi-country model resulting from these data may classify health status in a novel country in future applications. It should be noted that, there was some country-specific fitting as the AUCs of the LOCOCV model were lower than the multi-country model (Fig. 2b). The high classification capability suggests similarities between the participating countries in terms of metabolomic profile. The multi-country model can be used to classify chicken health in any of the participating countries using DI-MS data.

To enable potential application of the classification model without relying on full mass spectrometry profiles, we aimed to determine a compact group of metabolites that can achieve a good classification. To do so, we ranked all m/z values based on two common univariate analyses often used in omics studies—the t-test and fold-change analyses. By combining these two into a volcano plot and giving each m/z value a corresponding combined ‘*V-score*’, we could rank m/z values in both magnitude and significance of the effect. The advantage of this approach is that the high-ranking m/z values *each* differ in abundance between chicken health groups, facilitating interpretation later on.

In order to find the minimal number of high-ranked m/z values necessary to build a good classifying model, we built cross-validated RF models classifying health from a limited number of m/z values, starting at the top V-score-ranked to the lowest ranking m/z values, measuring classification capability of the model at each iteration of adding additional m/z values. This resulted in a compact 25-m/z signature that could classify health accurately. Using these 25 m/z values resulted in a better classifying model than using 25 random m/z values, and this compact m/z signature could potentially be used to develop a per metabolite panel in future applications. This would require identification of which compounds these 25 m/z values represent, and, if any m/z values cannot be identified, re-evaluating classification capability with alternative or without the missing signature members.

In the expanded m/z signature, which rather than minimizing the number of m/z used, maximised the number of m/z containing predictive information, we aimed to gain biological insights through enrichment analysis. Within pathways of interest that were enriched in this m/z signature, we found changes to pathways involved in amino acid (AA) metabolism (Fig. 6).

Within the amino acid group, levels of m/z predicted to match the branched chain amino acids (BCAAs) leucine and valine were increased which have been previously associated with fasting in humans (Ding et al., 2021). Furthermore, changes to phenylalanine metabolism, specifically lower levels of the predicted pathway members in unhealthy chickens, have also been found to connect to fasting in chickens by Wang et al. (2021). This may suggest that unhealthy animals were not consuming as much feed as the healthy animals, possibly due to illness, leading to metabolic adaptations in ways that have previously been connected to fasting (Ding et al., 2021; Wang et al., 2021).

Glutamine and serine were predicted to increase in the unhealthy chickens. These amino acids are connected to nitrogen excretion by playing an important role in uric acid synthesis, and threonine can also be degraded into serine. Furthermore, we observed potential increased levels of creatine and creatinine which are also nitrogen excretion products (van Milgen, 2021). These changes are indicative of excess protein intake. Feed composition is determined based on healthy animals, and the same feed may cause imbalances in unhealthy animals, as unhealthy chickens may have different requirements in terms of amino acid intake. Given this hypothesis, this signal is more likely to be a consequence than a cause of low health.

As we both saw a signal associated to fasting (increased BCAA’s), and with excess protein intake, a contradiction is present in the results. We hypothesise that the signal corresponding to fasting is the end-state of a process that started with a decrease in digestive efficiency, excess protein flow into the caeca and thus increased protein fermentation, leading to local inflammation and overall reduction in health and a further decrease in digestive efficiency due to the body needing to manage the disease process. Subsequently, these sick chickens likely ate less and could not compete with healthy, more dominant broilers in terms of feeding behaviour.

Furthermore, threonine has been connected to increased gut health due to being abundantly present in the chicken mucin (MUC2) protein which is an essential to form the gut mucus protective layer (Jiang et al., 2013). Additionally, Zhang et al. (2017) observed that threonine is required to synthesise both mucin and immunoglobulin in LPS-challenged broilers, suggesting that threonine also plays a role in the immune response. Cysteine may also have an immunoregulatory role (Qaisrani et al., 2018). Further research into this connection may elucidate the significance of increased threonine and cysteine levels in the blood of unhealthy chickens.

Metzler-Zebeli et al. (2019) investigated the serum metabolome in the context of chicken feed efficiency using the residual feed intake (RFI), where a high RFI corresponds to poor feed efficiency and thus likely unhealthy chickens in our situation. They noticed significant metabolomic differences between chickens with low or high feed efficiency, and that ‘low efficiency’/unhealthy animals at equal feed amounts showed increased relative serum levels of proline, serine, leucine, isoleucine, carnosine and valine. This corresponds to the changes in m/z values predicted to annotate for serine, leucine, valine and carnosine metabolites in our dataset, which would suggest that unhealthy animals were indeed less ‘feed efficient’. Their study suggests physiological differences in feed efficiency. On top of that, our data suggests that physiology is different between healthy and unhealthy animals, which may result in lower feed efficiency in unhealthy birds.

Similarly, Beauclercq et al. (2018) investigated the connection between chicken digestive efficiency and the metabolome. In serum, five metabolites were connected to a lower digestive efficiency—proline, valine, isoleucine, methionine and glutamine. In our results, we also observed higher levels of m/z values corresponding to (iso)leucine, valine, methionine and glutamine in the unhealthy group. These increases in blood amino acid levels may be due to altered protein metabolism and amino acid utilization in the low efficiency / unhealthy birds. In our data, this suggests that chickens in the ‘unhealthy’ group have a lower digestive efficiency as compared to chickens in the ‘healthy’ group.

We also observed an increase in m/z values matching putrescine in unhealthy chickens. Putrescine is produced by *E. coli* and other bacteria from dietary ornithine, and among other biogenic amines is also produced by many colonic bacterium species, including *Lactobacilli* (Chander et al., 1989), *Streptococci* (Babu et al., 1986), *Bacteroides* and *Clostridia* (Allison & Macfarlane, 1989), Furthermore, putrescine is suspected to be connected to decreased energy supply to colonocytes (Villodre Tudela et al., 2015) and thus decreased gut health as reviewed by Gilbert et al, (2018). Altogether, this suggests that excessive protein fermentation is involved in the chicken’s reduced health.

The protein fermentation in unhealthy chickens seems to occur despite chickens being fed the same feed within a farm. Therefore, the protein fermentation is likely attributable to physiological differences, such as the microbiome, including the presence of pathogenic bacteria, differences in digestive capacity or subtle differences in genetics. If indeed protein fermentation is the underlying cause of our classifying signature for unhealthy chickens, then interventions, nutritional or otherwise, could be specifically targeted to this.

By sharing the raw data and metadata of the samples we have collected, we facilitate future research. On the open data platform *MetaboLights*, only two datasets featuring chicken blood samples are currently hosted, together numbering almost 300 samples. Addition of our dataset of 197 samples would significantly increase the number of openly available chicken blood samples for fellow researchers.

In conclusion, we compared and characterised the metabolomes of healthy and unhealthy chickens using unbiased, untargeted mass spectrometry metabolomics. Future work may include a more fine-grained phenotyping effort that would enable signature discovery characterising specific sub-components of the health phenotype. To our knowledge, this is the first time such an approach was used to find a classifying signature for chicken health and to gain biological insights from this signature. The obtained compact m/z signature may be used in the future to aid development of a testing panel to selectively treat groups of chickens. Furthermore, the expanded signature and enriched pathways and the metabolites within those pathways could guide dietary adjustments and illuminate potential causes and metabolic consequences of low chicken health.

## Supplementary Information

Below is the link to the electronic supplementary material.

Supplementary file1 (DOCX 426 KB)

## Data Availability

The data reported in this paper is available via *MetaboLights* study identifier MTBLS5065.
