# Supplementary material for: Multi-country metabolic signature discovery for chicken health classification
Source: Metabolomics. 2023 Feb 2;19(2):9. doi: 10.1007/s11306-023-01973-4 (PMC9895029; doi:10.1007/s11306-023-01973-4)
Supplement: Supplementary file 1 — Supplementary file1 (DOCX 426 KB) [file 11306_2023_1973_MOESM1_ESM.docx]

*Supplementary materials for*

Multi-country metabolic signature discovery for chicken health classification

Joanna C. Wolthuis^1,2^ , Stefanía Magnúsdóttir^1^, Edwin Stigter^1^, Yuen Fung Tang^1^, Judith Jans^1^, Myrthe Gilbert^3^, Bart van der Hee^4^, Pim Langhout^5^, Walter Gerrits^3^, Arie Kies^4^, Jeroen de Ridder^1,2^ *,Saskia van Mil^1^*

*^1^Center for Molecular Medicine, University Medical Center Utrecht and Utrecht University, Utrecht, The Netherlands*

*^2^Oncode Institute, Utrecht, The Netherlands*

*^3^Animal Nutrition Group, Department of Animal Sciences, Wageningen University and Research, Wageningen, The Netherlands*

*^4^Host-Microbe Interactomics, Department of Animal Sciences, Wageningen University and Research, Wageningen, The Netherlands*

*^5^DSM Nutritional Products, Animal Nutrition and Health, Kaiseraugst, Switzerland.*

**Joint senior authors*

**
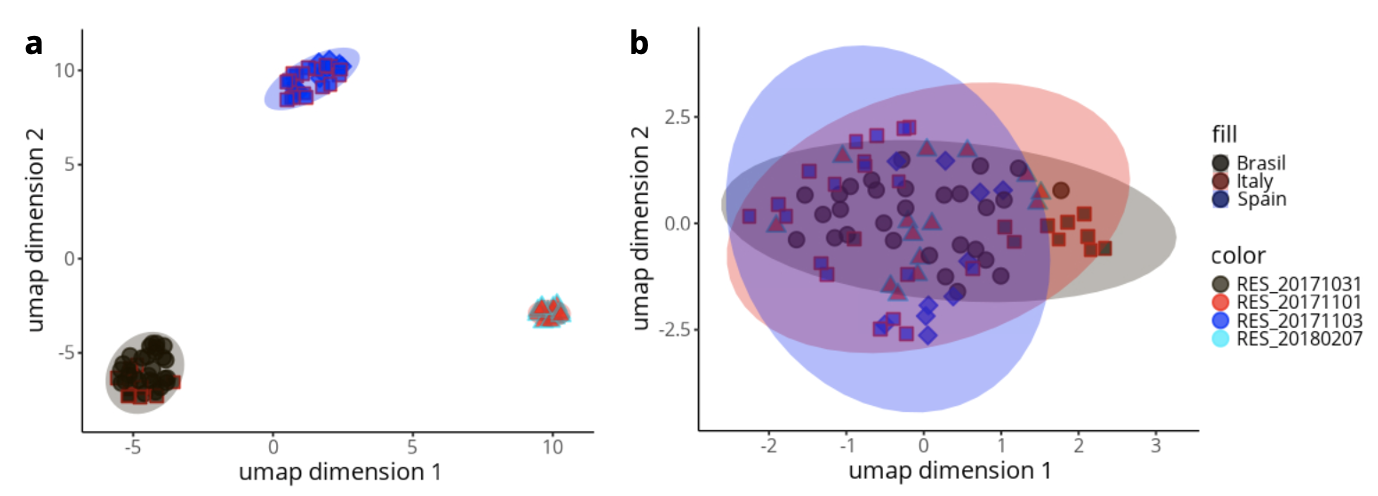
**

**Figure S1:** UMAP visualizations of original (a) and waveICA batch corrected (b) data. Fill represents country, and outline color represents day-of-run batch.

## Table S1 - Adducts used

| Name | Mode | Charge | xM | AddEx | RemEx | Nelec |
| --- | --- | --- | --- | --- | --- | --- |
| [M+H]1+ | + | 1 | 1 | H1 |  | -1 |
| [M+Na]1+ | + | 1 | 1 | Na1 |  | -1 |
| [M-H]1- | - | -1 | 1 |  | H1 | 1 |
| [M+Cl]1- | - | -1 | 1 | Cl1 |  | 1 |
| [M+NaCl+H]1+ | + | 1 | 1 | Na1Cl1H1 |  | -1 |
| [M+NaCl-H]1- | - | -1 | 1 | Na1Cl1 | H1 | 1 |

**Addex = Added atoms*

**RemEx = removed atoms*

**xM = ‘copies’ of metabolite (dimers, etc.)*

**Nelec = electron amount*

## Table S2 – G*allus gallus* enrichment results

| **Pathway** | **Pathway total** | **Hits.total** | **Hits.sig** | **Expected** | **FET** | **EASE** | **Gamma** | **Emp.Hits** |
| --- | --- | --- | --- | --- | --- | --- | --- | --- |
| D-Amino acid metabolism | 63 | 55 | 47 | 19.397 | 0.0037162 | 0.0093547 | 0.14721 | 0 |
| Glycine, serine and threonine metabolism | 44 | 35 | 31 | 13.547 | 0.0061675 | 0.018848 | 0.14763 | 0 |
| Phenylalanine metabolism | 49 | 35 | 31 | 15.086 | 0.0061675 | 0.018848 | 0.14763 | 0 |
| Valine, leucine and isoleucine degradation | 36 | 14 | 14 | 11.084 | 0.0054753 | 0.039714 | 0.14857 | 0 |
| Arginine and proline metabolism | 71 | 53 | 43 | 21.86 | 0.033031 | 0.063789 | 0.14969 | 0 |
| Butanoate metabolism | 46 | 28 | 24 | 14.163 | 0.036845 | 0.090563 | 0.15096 | 0 |
| Cysteine and methionine metabolism | 64 | 35 | 28 | 19.704 | 0.10667 | 0.19401 | 0.15623 | 0 |
| Thiamine metabolism | 26 | 12 | 11 | 8.0049 | 0.074567 | 0.22885 | 0.15816 | 0 |
| Ubiquinone and other terpenoid-quinone biosynthesis | 56 | 23 | 19 | 17.241 | 0.11464 | 0.23418 | 0.15846 | 0 |
| Purine metabolism | 99 | 26 | 21 | 30.48 | 0.1373 | 0.25789 | 0.15983 | 0 |
| Porphyrin metabolism | 130 | 11 | 10 | 40.025 | 0.10063 | 0.28722 | 0.16158 | 0 |
| Sulfur metabolism | 32 | 14 | 12 | 9.8522 | 0.14282 | 0.32485 | 0.16393 | 0 |
| Propanoate metabolism | 37 | 21 | 17 | 11.392 | 0.17286 | 0.32525 | 0.16396 | 0 |
| Fatty acid biosynthesis | 12 | 9 | 8 | 3.6946 | 0.17974 | 0.43668 | 0.17176 | 0 |
| Glutathione metabolism | 29 | 12 | 10 | 8.9286 | 0.23093 | 0.46235 | 0.17377 | 0 |
| Glycerophospholipid metabolism | 28 | 15 | 12 | 8.6207 | 0.27029 | 0.4819 | 0.17536 | 0 |
| Lysine degradation | 44 | 31 | 23 | 13.547 | 0.344 | 0.49388 | 0.17637 | 0 |
| Metabolism of xenobiotics by cytochrome P450 | 121 | 31 | 23 | 37.254 | 0.344 | 0.49388 | 0.17637 | 0 |
| Pantothenate and CoA biosynthesis | 28 | 21 | 16 | 8.6207 | 0.32857 | 0.51127 | 0.17787 | 0 |
| Alanine, aspartate and glutamate metabolism | 28 | 21 | 16 | 8.6207 | 0.32857 | 0.51127 | 0.17787 | 0 |
| Taurine and hypotaurine metabolism | 23 | 14 | 11 | 7.0813 | 0.32756 | 0.55246 | 0.18164 | 0 |
| Riboflavin metabolism | 24 | 4 | 4 | 7.3892 | 0.22775 | 0.63465 | 0.19027 | 0 |
| Sphingolipid metabolism | 11 | 4 | 4 | 3.3867 | 0.22775 | 0.63465 | 0.19027 | 0 |
| Pentose phosphate pathway | 35 | 25 | 18 | 10.776 | 0.47269 | 0.63858 | 0.19073 | 0 |
| Tyrosine metabolism | 76 | 62 | 43 | 23.399 | 0.54654 | 0.64846 | 0.1919 | 0 |
| Pyrimidine metabolism | 62 | 33 | 23 | 19.089 | 0.55631 | 0.69367 | 0.19771 | 0 |
| Folate biosynthesis | 57 | 6 | 5 | 17.549 | 0.40092 | 0.72658 | 0.20248 | 0 |
| Biotin metabolism | 16 | 6 | 5 | 4.9261 | 0.40092 | 0.72658 | 0.20248 | 0 |
| Fatty acid degradation | 39 | 3 | 3 | 12.007 | 0.32988 | 0.77192 | 0.21003 | 0 |
| Phosphonate and phosphinate metabolism | 52 | 25 | 17 | 16.01 | 0.64243 | 0.78223 | 0.21195 | 0 |
| Terpenoid backbone biosynthesis | 39 | 16 | 11 | 12.007 | 0.63121 | 0.8027 | 0.21603 | 0 |
| alpha-Linolenic acid metabolism | 43 | 30 | 20 | 13.239 | 0.69614 | 0.81332 | 0.21831 | 0 |
| Caffeine metabolism | 22 | 5 | 4 | 6.7734 | 0.51006 | 0.82437 | 0.22081 | 0 |
| Biosynthesis of unsaturated fatty acids | 69 | 18 | 12 | 21.244 | 0.69424 | 0.83938 | 0.22446 | 0 |
| beta-Alanine metabolism | 32 | 22 | 14 | 9.8522 | 0.78991 | 0.89259 | 0.24073 | 1 |
| Ether lipid metabolism | 2 | 2 | 2 | 0.61576 | 0.47762 | 0.9043 | 0.24534 | 0 |
| Lipoic acid metabolism | 6 | 2 | 2 | 1.8473 | 0.47762 | 0.9043 | 0.24534 | 0 |
| Glycerolipid metabolism | 14 | 11 | 7 | 4.3103 | 0.771 | 0.9114 | 0.2484 | 0 |
| Nicotinate and nicotinamide metabolism | 55 | 40 | 25 | 16.933 | 0.86335 | 0.92166 | 0.25326 | 3 |
| Citrate cycle (TCA cycle) | 16 | 10 | 6 | 4.9261 | 0.83533 | 0.94599 | 0.26777 | 1 |
| Histidine metabolism | 47 | 29 | 17 | 14.47 | 0.9229 | 0.96391 | 0.2832 | 1 |
| Glyoxylate and dicarboxylate metabolism | 61 | 34 | 20 | 18.781 | 0.93158 | 0.96654 | 0.28606 | 2 |
| Oxidative phosphorylation | 13 | 3 | 2 | 4.0025 | 0.7731 | 0.97048 | 0.29078 | 1 |
| Linoleic acid metabolism | 27 | 27 | 15 | 8.3128 | 0.95712 | 0.98225 | 0.30958 | 0 |
| Nitrogen metabolism | 17 | 6 | 3 | 5.234 | 0.92293 | 0.98754 | 0.32236 | 0 |
| Inositol phosphate metabolism | 38 | 21 | 11 | 11.7 | 0.96879 | 0.98921 | 0.32752 | 0 |
| Neomycin, kanamycin and gentamicin biosynthesis | 76 | 9 | 4 | 23.399 | 0.97137 | 0.99493 | 0.35385 | 0 |
| Vitamin B6 metabolism | 29 | 24 | 12 | 8.9286 | 0.98612 | 0.99542 | 0.35729 | 0 |
| Tryptophan metabolism | 83 | 56 | 31 | 25.554 | 0.99126 | 0.99586 | 0.36073 | 1 |
| Pyruvate metabolism | 29 | 17 | 8 | 8.9286 | 0.98499 | 0.99601 | 0.36193 | 1 |
| Drug metabolism - other enzymes | 61 | 16 | 7 | 18.781 | 0.99153 | 0.99806 | 0.38579 | 1 |
| Drug metabolism - cytochrome P450 | 100 | 27 | 13 | 30.788 | 0.99392 | 0.99807 | 0.38596 | 0 |
| Ascorbate and aldarate metabolism | 56 | 44 | 22 | 17.241 | 0.99794 | 0.99923 | 0.41497 | 0 |
| Galactose metabolism | 45 | 33 | 15 | 13.855 | 0.99887 | 0.99966 | 0.43999 | 0 |
| Glycolysis / Gluconeogenesis | 28 | 16 | 6 | 8.6207 | 0.99811 | 0.99966 | 0.44029 | 0 |
| Primary bile acid biosynthesis | 47 | 22 | 9 | 14.47 | 0.99862 | 0.99968 | 0.44208 | 0 |
| Steroid biosynthesis | 56 | 9 | 2 | 17.241 | 0.99949 | 0.99998 | 0.51279 | 0 |
| Pentose and glucuronate interconversions | 55 | 43 | 18 | 16.933 | 0.99996 | 0.99999 | 0.53238 | 0 |
| Amino sugar and nucleotide sugar metabolism | 110 | 37 | 14 | 33.867 | 0.99998 | 1 | 0.56079 | 0 |
| Starch and sucrose metabolism | 24 | 12 | 2 | 7.3892 | 0.99998 | 1 | 0.59681 | 0 |
| Fructose and mannose metabolism | 48 | 35 | 8 | 14.778 | 1 | 1 | 0.74057 | 0 |
| Steroid hormone biosynthesis | 99 | 54 | 12 | 30.48 | 1 | 1 | 0.85038 | 0 |
| Fatty acid elongation | 28 | 1 | 1 | 8.6207 | 0.69124 | 1 | 1 | 0 |
| Selenocompound metabolism | 22 | 1 | 1 | 6.7734 | 0.69124 | 1 | 1 | 0 |
| Mannose type O-glycan biosynthesis | 3 | 1 | 1 | 0.92365 | 0.69124 | 1 | 1 | 0 |

## Table S3 – *Microbial metabolism in diverse environments* enrichment results

| **Pathway** | **Pathway total** | **Hits.total** | **Hits.sig** | **Expected** | **FET** | **EASE** | **Gamma** | **Emp.Hits** |
| --- | --- | --- | --- | --- | --- | --- | --- | --- |
| Glycine, serine and threonine metabolism | 44 | 35 | 31 | 16.271 | 0.0042819 | 0.013589 | 0.14641 | 0 |
| Phenylalanine metabolism | 59 | 45 | 38 | 21.819 | 0.009422 | 0.022842 | 0.14682 | 0 |
| Ethylbenzene degradation | 14 | 11 | 11 | 5.1773 | 0.014361 | 0.088023 | 0.14985 | 0 |
| Lysine biosynthesis | 30 | 19 | 17 | 11.094 | 0.031154 | 0.096121 | 0.15024 | 0 |
| Purine metabolism | 97 | 25 | 21 | 35.871 | 0.060553 | 0.1383 | 0.15233 | 0 |
| Naphthalene degradation | 60 | 45 | 35 | 22.188 | 0.1022 | 0.17294 | 0.15413 | 0 |
| Caprolactam degradation | 24 | 18 | 15 | 8.8753 | 0.12486 | 0.26847 | 0.15948 | 0 |
| Sulfur metabolism | 32 | 14 | 12 | 11.834 | 0.12637 | 0.29676 | 0.1612 | 0 |
| Xylene degradation | 38 | 34 | 26 | 14.053 | 0.19352 | 0.31093 | 0.16208 | 0 |
| Lysine degradation | 44 | 31 | 23 | 16.271 | 0.30249 | 0.4466 | 0.17158 | 0 |
| Dioxin degradation | 66 | 21 | 16 | 24.407 | 0.29518 | 0.47237 | 0.17364 | 0 |
| Tyrosine metabolism | 76 | 62 | 43 | 28.105 | 0.48132 | 0.58458 | 0.18389 | 0 |
| Pentose phosphate pathway | 35 | 25 | 18 | 12.943 | 0.43197 | 0.59824 | 0.18531 | 0 |
| Toluene degradation | 47 | 28 | 20 | 17.381 | 0.44299 | 0.59975 | 0.18547 | 0 |
| Furfural degradation | 10 | 7 | 6 | 3.6981 | 0.29145 | 0.60312 | 0.18583 | 0 |
| Bisphenol degradation | 25 | 20 | 14 | 9.2452 | 0.53834 | 0.71224 | 0.19944 | 0 |
| Aminobenzoate degradation | 86 | 58 | 39 | 31.803 | 0.62654 | 0.72225 | 0.20092 | 0 |
| Phosphonate and phosphinate metabolism | 52 | 25 | 17 | 19.23 | 0.60371 | 0.75011 | 0.20535 | 0 |
| Carbon fixation in photosynthetic organisms | 22 | 16 | 11 | 8.1357 | 0.59986 | 0.77832 | 0.21034 | 0 |
| Styrene degradation | 24 | 21 | 14 | 8.8753 | 0.66004 | 0.80482 | 0.21561 | 0 |
| Caffeine metabolism | 22 | 5 | 4 | 8.1357 | 0.49116 | 0.81115 | 0.21697 | 0 |
| Benzoate degradation | 72 | 34 | 22 | 26.626 | 0.74127 | 0.84024 | 0.22383 | 0 |
| Nicotinate and nicotinamide metabolism | 55 | 40 | 25 | 20.339 | 0.83407 | 0.90115 | 0.24323 | 0 |
| Chlorocyclohexane and chlorobenzene degradation | 83 | 26 | 16 | 30.694 | 0.83109 | 0.91197 | 0.24785 | 0 |
| Citrate cycle (TCA cycle) | 16 | 10 | 6 | 5.9169 | 0.81855 | 0.93815 | 0.26174 | 0 |
| Carbon fixation pathways in prokaryotes | 44 | 17 | 10 | 16.271 | 0.86405 | 0.94332 | 0.26514 | 0 |
| Glyoxylate and dicarboxylate metabolism | 61 | 34 | 20 | 22.558 | 0.91524 | 0.95686 | 0.27567 | 0 |
| Nitrotoluene degradation | 27 | 15 | 8 | 9.9848 | 0.93311 | 0.97787 | 0.30079 | 0 |
| Nitrogen metabolism | 17 | 6 | 3 | 6.2867 | 0.91512 | 0.98571 | 0.31679 | 0 |
| Inositol phosphate metabolism | 38 | 21 | 11 | 14.053 | 0.96189 | 0.98628 | 0.31825 | 0 |
| Vitamin B6 metabolism | 29 | 24 | 12 | 10.724 | 0.98239 | 0.99395 | 0.34714 | 2 |
| Methane metabolism | 88 | 38 | 20 | 32.543 | 0.98722 | 0.99464 | 0.35131 | 1 |
| Pyruvate metabolism | 29 | 17 | 8 | 10.724 | 0.98162 | 0.9949 | 0.35305 | 0 |
| Ascorbate and aldarate metabolism | 56 | 44 | 22 | 20.709 | 0.99702 | 0.99883 | 0.40166 | 0 |
| Fluorobenzoate degradation | 32 | 13 | 5 | 11.834 | 0.99415 | 0.99897 | 0.40553 | 0 |
| Steroid degradation | 18 | 13 | 5 | 6.6565 | 0.99415 | 0.99897 | 0.40553 | 0 |
| Glycolysis / Gluconeogenesis | 28 | 16 | 6 | 10.355 | 0.99759 | 0.99955 | 0.43131 | 0 |
| Atrazine degradation | 23 | 7 | 2 | 8.5055 | 0.99492 | 0.99968 | 0.44128 | 0 |
| Chloroalkane and chloroalkene degradation | 43 | 15 | 4 | 15.902 | 0.99985 | 0.99998 | 0.52402 | 0 |
| Polycyclic aromatic hydrocarbon degradation | 106 | 56 | 24 | 39.199 | 0.99999 | 1 | 0.55747 | 0 |
| Fructose and mannose metabolism | 48 | 35 | 8 | 17.751 | 1 | 1 | 0.73266 | 0 |

## Table S4 *– D-amino acid metabolism* hits

| **m/z** | **compoundname** | **identifier** | **adduct** | **Δppm** | **in sig** |
| --- | --- | --- | --- | --- | --- |
| 89.10727 | Putrescine | C00134 | [M+H]1+ | 0.0724081323571241 | yes |
| 213.08454 | LL-2,6-Diaminoheptanedioate | C00666 | [M+Na]1+ | 0.380506680622534 | yes |
| 213.08454 | meso-2,6-Diaminoheptanedioate | C00680 | [M+Na]1+ | 0.380506680622534 | yes |
| 197.10092 | L-Arginine | C00062 | [M+Na]1+ | 0.00547988822379961 | yes |
| 197.10092 | D-Arginine | C00792 | [M+Na]1+ | 0.00547988822379961 | yes |
| 188.06823 | L-Phenylalanine | C00079 | [M+Na]1+ | 0.0474291111941984 | yes |
| 188.06823 | D-Phenylalanine | C02265 | [M+Na]1+ | 0.0474291111941984 | yes |
| 183.07413 | D-Alanyl-D-alanine | C00993 | [M+Na]1+ | 0.59494975612385 | yes |
| 175.11895 | L-Arginine | C00062 | [M+H]1+ | 0.151052133377224 | yes |
| 175.11895 | D-Arginine | C00792 | [M+H]1+ | 0.151052133377224 | yes |
| 172.04024 | L-Methionine | C00073 | [M+Na]1+ | 0.109973742331224 | yes |
| 172.04024 | D-Methionine | C00855 | [M+Na]1+ | 0.109973742331224 | yes |
| 169.05838 | L-Glutamine | C00064 | [M+Na]1+ | 0.348518121424308 | yes |
| 169.05838 | D-Glutamine | C00819 | [M+Na]1+ | 0.348518121424308 | yes |
| 168.06315 | 6-Amino-2-oxohexanoate | C03239 | [M+Na]1+ | 0.172077632717906 | yes |
| 168.06315 | 2-Amino-5-oxohexanoate | C05825 | [M+Na]1+ | 0.172077632717906 | yes |
| 165.05463 | Phenylpyruvate | C00166 | [M+H]1+ | 0.324425367556742 | yes |
| 164.00832 | L-Serine | C00065 | [M+NaCl+H]1+ | 1.09032328961906 | yes |
| 164.00832 | D-Serine | C00740 | [M+NaCl+H]1+ | 1.09032328961906 | yes |
| 161.09202 | D-Alanyl-D-alanine | C00993 | [M+H]1+ | 0.350433814175288 | yes |
| 156.02673 | L-Aspartate | C00049 | [M+Na]1+ | 0.057169108092992 | yes |
| 156.02673 | D-Aspartate | C00402 | [M+Na]1+ | 0.057169108092992 | yes |
| 154.04745 | 5-Amino-2-oxopentanoic acid | C01110 | [M+Na]1+ | 0.187733772932394 | yes |
| 154.04745 | Hydroxyproline | C01157 | [M+Na]1+ | 0.187733772932394 | yes |
| 154.04745 | 2-Amino-4-oxopentanoic acid | C03341 | [M+Na]1+ | 0.187733772932394 | yes |
| 154.04745 | cis-4-Hydroxy-D-proline | C03440 | [M+Na]1+ | 0.187733772932394 | yes |
| 152.03183 | 5-Oxo-D-proline | C02237 | [M+Na]1+ | 0.0586713257426934 | yes |
| 152.03183 | 1-Pyrroline-4-hydroxy-2-carboxylate | C04282 | [M+Na]1+ | 0.0586713257426934 | yes |
| 148.06044 | L-Glutamate | C00025 | [M+H]1+ | 0.246197370448538 | yes |
| 148.06044 | D-Glutamate | C00217 | [M+H]1+ | 0.246197370448538 | yes |
| 148.06044 | 2-Oxo-4-hydroxy-5-aminovalerate | C05941 | [M+H]1+ | 0.246197370448538 | yes |
| 147.07644 | L-Glutamine | C00064 | [M+H]1+ | 0.432074022242217 | yes |
| 147.07644 | D-Glutamine | C00819 | [M+H]1+ | 0.432074022242217 | yes |
| 142.04751 | L-Threonine | C00188 | [M+Na]1+ | 0.625987101004006 | yes |
| 142.04751 | D-Threonine | C00820 | [M+Na]1+ | 0.625987101004006 | yes |
| 136.03696 | 1-Pyrroline-2-carboxylate | C03564 | [M+Na]1+ | 0.286098049926041 | yes |
| 134.04478 | L-Aspartate | C00049 | [M+H]1+ | 0.0264680878825942 | yes |
| 134.04478 | D-Aspartate | C00402 | [M+H]1+ | 0.0264680878825942 | yes |
| 132.06558 | 5-Amino-2-oxopentanoic acid | C01110 | [M+H]1+ | 0.784064318807088 | yes |
| 132.06558 | Hydroxyproline | C01157 | [M+H]1+ | 0.784064318807088 | yes |
| 132.06558 | 2-Amino-4-oxopentanoic acid | C03341 | [M+H]1+ | 0.784064318807088 | yes |
| 132.06558 | cis-4-Hydroxy-D-proline | C03440 | [M+H]1+ | 0.784064318807088 | yes |
| 130.04992 | 5-Oxo-D-proline | C02237 | [M+H]1+ | 0.334855330949206 | yes |
| 130.04992 | 1-Pyrroline-4-hydroxy-2-carboxylate | C04282 | [M+H]1+ | 0.334855330949206 | yes |
| 122.02695 | L-Cysteine | C00097 | [M+H]1+ | 0.216772532678153 | yes |
| 122.02695 | D-Cysteine | C00793 | [M+H]1+ | 0.216772532678153 | yes |
| 118.08619 | 5-Aminopentanoate | C00431 | [M+H]1+ | 0.732110088377933 | yes |
| 114.05497 | 1-Pyrroline-2-carboxylate | C03564 | [M+H]1+ | 0.056570011936813 | yes |
| 106.04983 | L-Serine | C00065 | [M+H]1+ | 0.438021362208951 | yes |
| 106.04983 | D-Serine | C00740 | [M+H]1+ | 0.438021362208951 | yes |
| 98.02124 | Glycine | C00037 | [M+Na]1+ | 0.193018462224627 | no |
| 90.05496 | L-Alanine | C00041 | [M+H]1+ | 0.182689448949031 | no |
| 90.05496 | D-Alanine | C00133 | [M+H]1+ | 0.182689448949031 | no |
| 89.02327 | Pyruvate | C00022 | [M+H]1+ | 0.0724764549128879 | no |
| 88.04044- | L-Alanine | C00041 | [M-H]1- | 0.186869704459861 | no |
| 88.04044- | D-Alanine | C00133 | [M-H]1- | 0.186869704459861 | no |
| 87.00878- | Pyruvate | C00022 | [M-H]1- | 0.648809131696845 | no |
| 76.03930 | Glycine | C00037 | [M+H]1+ | 0.309680770274971 | no |
| 74.02475- | Glycine | C00037 | [M-H]1- | 0.357341173141285 | no |
| 260.12407 | Linatine | C05939 | [M+H]1+ | 0.0248038983317889 | no |
| 249.06098 | LL-2,6-Diaminoheptanedioate | C00666 | [M+NaCl+H]1+ | 1.28009650890843 | no |
| 249.06098 | meso-2,6-Diaminoheptanedioate | C00680 | [M+NaCl+H]1+ | 1.28009650890843 | no |
| 231.06303- | L-Arginine | C00062 | [M+NaCl-H]1- | 0.0688898999751942 | no |
| 231.06303- | D-Arginine | C00792 | [M+NaCl-H]1- | 0.0688898999751942 | no |
| 230.07863 | N-Acetyl-D-phenylalanine | C05620 | [M+Na]1+ | 0.395865061358075 | no |
| 222.03037- | L-Phenylalanine | C00079 | [M+NaCl-H]1- | 0.108463049392888 | no |
| 222.03037- | D-Phenylalanine | C02265 | [M+NaCl-H]1- | 0.108463049392888 | no |
| 212.02080- | L-Histidine | C00135 | [M+NaCl-H]1- | 0.216572661766044 | no |
| 212.02080- | D-Histidine | C06419 | [M+NaCl-H]1- | 0.216572661766044 | no |
| 209.08107- | L-Arginine | C00062 | [M+Cl]1- | 0.149654433102731 | no |
| 209.08107- | D-Arginine | C00792 | [M+Cl]1- | 0.149654433102731 | no |
| 206.08238- | N-Acetyl-D-phenylalanine | C05620 | [M-H]1- | 0.759172574604006 | no |
| 204.00452- | L-Glutamate | C00025 | [M+NaCl-H]1- | 0.127045758591122 | no |
| 204.00452- | D-Glutamate | C00217 | [M+NaCl-H]1- | 0.127045758591122 | no |
| 204.00452- | 2-Oxo-4-hydroxy-5-aminovalerate | C05941 | [M+NaCl-H]1- | 0.127045758591122 | no |
| 203.05650- | L-Lysine | C00047 | [M+NaCl-H]1- | 1.70355496630605 | no |
| 203.05650- | D-Lysine | C00739 | [M+NaCl-H]1- | 1.70355496630605 | no |
| 203.05650- | (2R,5S)-2,5-Diaminohexanoate | C05161 | [M+NaCl-H]1- | 1.70355496630605 | no |
| 203.02063- | L-Glutamine | C00064 | [M+NaCl-H]1- | 0.906716184509899 | no |
| 203.02063- | D-Glutamine | C00819 | [M+NaCl-H]1- | 0.906716184509899 | no |
| 200.04847- | L-Phenylalanine | C00079 | [M+Cl]1- | 0.343467215720056 | no |
| 200.04847- | D-Phenylalanine | C02265 | [M+Cl]1- | 0.343467215720056 | no |
| 195.05406- | D-Alanyl-D-alanine | C00993 | [M+Cl]1- | 0.724362820286109 | no |
| 192.00331 | L-Aspartate | C00049 | [M+NaCl+H]1+ | 0.462607082071401 | no |
| 192.00331 | D-Aspartate | C00402 | [M+NaCl+H]1+ | 0.462607082071401 | no |
| 190.03894- | L-Histidine | C00135 | [M+Cl]1- | 0.203695574130411 | no |
| 190.03894- | D-Histidine | C06419 | [M+Cl]1- | 0.203695574130411 | no |
| 189.98896- | L-Aspartate | C00049 | [M+NaCl-H]1- | 0.600466948122549 | no |
| 189.98896- | D-Aspartate | C00402 | [M+NaCl-H]1- | 0.600466948122549 | no |
| 189.08818- | LL-2,6-Diaminoheptanedioate | C00666 | [M-H]1- | 0.298549020744608 | no |
| 189.08818- | meso-2,6-Diaminoheptanedioate | C00680 | [M-H]1- | 0.298549020744608 | no |
| 187.03669 | Phenylpyruvate | C00166 | [M+Na]1+ | 0.903137822780701 | no |
| 185.994- | 5-Oxo-D-proline | C02237 | [M+NaCl-H]1- | 0.290773309878564 | no |
| 185.994- | 1-Pyrroline-4-hydroxy-2-carboxylate | C04282 | [M+NaCl-H]1- | 0.290773309878564 | no |
| 182.02260- | L-Glutamate | C00025 | [M+Cl]1- | 0.00708653207810497 | no |
| 182.02260- | D-Glutamate | C00217 | [M+Cl]1- | 0.00708653207810497 | no |
| 182.02260- | 2-Oxo-4-hydroxy-5-aminovalerate | C05941 | [M+Cl]1- | 0.00708653207810497 | no |
| 181.07497- | L-Lysine | C00047 | [M+Cl]1- | 0.379456591929615 | no |
| 181.07497- | D-Lysine | C00739 | [M+Cl]1- | 0.379456591929615 | no |
| 181.07497- | (2R,5S)-2,5-Diaminohexanoate | C05161 | [M+Cl]1- | 0.379456591929615 | no |
| 181.03866- | L-Glutamine | C00064 | [M+Cl]1- | 0.8766640837935 | no |
| 181.03866- | D-Glutamine | C00819 | [M+Cl]1- | 0.8766640837935 | no |
| 180.04338- | 6-Amino-2-oxohexanoate | C03239 | [M+Cl]1- | 0.437172924675883 | no |
| 180.04338- | 2-Amino-5-oxohexanoate | C05825 | [M+Cl]1- | 0.437172924675883 | no |
| 176.00963- | L-Threonine | C00188 | [M+NaCl-H]1- | 0.477713014785921 | no |
| 176.00963- | D-Threonine | C00820 | [M+NaCl-H]1- | 0.477713014785921 | no |
| 174.08729 | 5-Guanidino-2-oxopentanoate | C03771 | [M+H]1+ | 0.0778225049743459 | no |
| 174.03035- | 5-Aminopentanoate | C00431 | [M+NaCl-H]1- | 0.0234562018067808 | no |
| 173.10444- | L-Arginine | C00062 | [M-H]1- | 0.0950414153970014 | no |
| 173.10444- | D-Arginine | C00792 | [M-H]1- | 0.0950414153970014 | no |
| 172.07280- | 5-Guanidino-2-oxopentanoate | C03771 | [M-H]1- | 0.444300848127818 | no |
| 172.01484- | L-Proline | C00148 | [M+NaCl-H]1- | 1.12828690236928 | no |
| 172.01484- | D-Proline | C00763 | [M+NaCl-H]1- | 1.12828690236928 | no |
| 170.04240 | L-Glutamate | C00025 | [M+Na]1+ | 0.12396961591201 | no |
| 170.04240 | D-Glutamate | C00217 | [M+Na]1+ | 0.12396961591201 | no |
| 170.04240 | 2-Oxo-4-hydroxy-5-aminovalerate | C05941 | [M+Na]1+ | 0.12396961591201 | no |
| 169.09477 | L-Lysine | C00047 | [M+Na]1+ | 0.289304684026967 | no |
| 169.09477 | D-Lysine | C00739 | [M+Na]1+ | 0.289304684026967 | no |
| 169.09477 | (2R,5S)-2,5-Diaminohexanoate | C05161 | [M+Na]1+ | 0.289304684026967 | no |
| 168.00693- | L-Aspartate | C00049 | [M+Cl]1- | 0.17088634984094 | no |
| 168.00693- | D-Aspartate | C00402 | [M+Cl]1- | 0.17088634984094 | no |
| 166.97514- | Oxaloacetate | C00036 | [M+Cl]1- | 0.965951632008112 | no |
| 166.08628 | L-Phenylalanine | C00079 | [M+H]1+ | 0.0213618427050163 | no |
| 166.08628 | D-Phenylalanine | C02265 | [M+H]1+ | 0.0213618427050163 | no |
| 166.02767- | 5-Amino-2-oxopentanoic acid | C01110 | [M+Cl]1- | 0.413847227994931 | no |
| 166.02767- | Hydroxyproline | C01157 | [M+Cl]1- | 0.413847227994931 | no |
| 166.02767- | 2-Amino-4-oxopentanoic acid | C03341 | [M+Cl]1- | 0.413847227994931 | no |
| 166.02767- | cis-4-Hydroxy-D-proline | C03440 | [M+Cl]1- | 0.413847227994931 | no |
| 164.99606- | 2,5-Dioxopentanoate | C00433 | [M+Cl]1- | 0.355827230036784 | no |
| 164.07162- | L-Phenylalanine | C00079 | [M-H]1- | 0.631114077014434 | no |
| 164.07162- | D-Phenylalanine | C02265 | [M-H]1- | 0.631114077014434 | no |
| 164.01209- | 5-Oxo-D-proline | C02237 | [M+Cl]1- | 0.540875315998384 | no |
| 164.01209- | 1-Pyrroline-4-hydroxy-2-carboxylate | C04282 | [M+Cl]1- | 0.540875315998384 | no |
| 163.04016- | Phenylpyruvate | C00166 | [M-H]1- | 0.83692319101227 | no |
| 161.99399- | L-Serine | C00065 | [M+NaCl-H]1- | 0.272121768186459 | no |
| 161.99399- | D-Serine | C00740 | [M+NaCl-H]1- | 0.272121768186459 | no |
| 159.07751- | D-Alanyl-D-alanine | C00993 | [M-H]1- | 0.0851654581494084 | no |
| 156.07676 | L-Histidine | C00135 | [M+H]1+ | 0.105410254474179 | no |
| 156.07676 | D-Histidine | C06419 | [M+H]1+ | 0.105410254474179 | no |
| 154.06221- | L-Histidine | C00135 | [M-H]1- | 0.0879379117073363 | no |
| 154.06221- | D-Histidine | C06419 | [M-H]1- | 0.0879379117073363 | no |
| 154.02766- | L-Threonine | C00188 | [M+Cl]1- | 0.381165895766923 | no |
| 154.02766- | D-Threonine | C00820 | [M+Cl]1- | 0.381165895766923 | no |
| 153.01605 | 2,5-Dioxopentanoate | C00433 | [M+Na]1+ | 1.49605161684512 | no |
| 152.04836- | 5-Aminopentanoate | C00431 | [M+Cl]1- | 0.271557739962053 | no |
| 150.05836 | L-Methionine | C00073 | [M+H]1+ | 0.55676943966477 | no |
| 150.05836 | D-Methionine | C00855 | [M+H]1+ | 0.55676943966477 | no |
| 148.04381- | L-Methionine | C00073 | [M-H]1- | 0.58396288916993 | no |
| 148.04381- | D-Methionine | C00855 | [M-H]1- | 0.58396288916993 | no |
| 147.11281 | L-Lysine | C00047 | [M+H]1+ | 0.228042065122294 | no |
| 147.11281 | D-Lysine | C00739 | [M+H]1+ | 0.228042065122294 | no |
| 147.11281 | (2R,5S)-2,5-Diaminohexanoate | C05161 | [M+H]1+ | 0.228042065122294 | no |
| 146.08120 | 6-Amino-2-oxohexanoate | C03239 | [M+H]1+ | 0.161197395661931 | no |
| 146.08120 | 2-Amino-5-oxohexanoate | C05825 | [M+H]1+ | 0.161197395661931 | no |
| 146.04590- | L-Glutamate | C00025 | [M-H]1- | 0.161236358046281 | no |
| 146.04590- | D-Glutamate | C00217 | [M-H]1- | 0.161236358046281 | no |
| 146.04590- | 2-Oxo-4-hydroxy-5-aminovalerate | C05941 | [M-H]1- | 0.161236358046281 | no |
| 145.99898- | L-Alanine | C00041 | [M+NaCl-H]1- | 0.451495681765135 | no |
| 145.99898- | D-Alanine | C00133 | [M+NaCl-H]1- | 0.451495681765135 | no |
| 145.09827- | L-Lysine | C00047 | [M-H]1- | 0.320142280115591 | no |
| 145.09827- | D-Lysine | C00739 | [M-H]1- | 0.320142280115591 | no |
| 145.09827- | (2R,5S)-2,5-Diaminohexanoate | C05161 | [M-H]1- | 0.320142280115591 | no |
| 145.06186- | L-Glutamine | C00064 | [M-H]1- | 0.25128652697169 | no |
| 145.06186- | D-Glutamine | C00819 | [M-H]1- | 0.25128652697169 | no |
| 145.01423- | 2-Oxoglutarate | C00026 | [M-H]1- | 0.044492812780726 | no |
| 144.96734- | Pyruvate | C00022 | [M+NaCl-H]1- | 0.0408223603541972 | no |
| 144.06662- | 6-Amino-2-oxohexanoate | C03239 | [M-H]1- | 0.0246268637193196 | no |
| 144.06662- | 2-Amino-5-oxohexanoate | C05825 | [M-H]1- | 0.0246268637193196 | no |
| 140.06821 | 5-Aminopentanoate | C00431 | [M+Na]1+ | 0.0791049661702838 | no |
| 140.01190- | L-Serine | C00065 | [M+Cl]1- | 0.723437857761805 | no |
| 140.01190- | D-Serine | C00740 | [M+Cl]1- | 0.723437857761805 | no |
| 138.05259 | L-Proline | C00148 | [M+Na]1+ | 0.499229380715066 | no |
| 138.05259 | D-Proline | C00763 | [M+Na]1+ | 0.499229380715066 | no |
| 133.09717 | L-Ornithine | C00077 | [M+H]1+ | 0.0484765452633677 | no |
| 133.09717 | D-Ornithine | C00515 | [M+H]1+ | 0.0484765452633677 | no |
| 133.09717 | (2R,4S)-2,4-Diaminopentanoate | C03943 | [M+H]1+ | 0.0484765452633677 | no |
| 132.03027- | L-Aspartate | C00049 | [M-H]1- | 0.35182909948323 | no |
| 132.03027- | D-Aspartate | C00402 | [M-H]1- | 0.35182909948323 | no |
| 131.98335- | Glycine | C00037 | [M+NaCl-H]1- | 0.030928833145277 | no |
| 131.08259- | L-Ornithine | C00077 | [M-H]1- | 0.255929555544666 | no |
| 131.08259- | D-Ornithine | C00515 | [M-H]1- | 0.255929555544666 | no |
| 131.08259- | (2R,4S)-2,4-Diaminopentanoate | C03943 | [M-H]1- | 0.255929555544666 | no |
| 131.03393 | 2,5-Dioxopentanoate | C00433 | [M+H]1+ | 0.408656818922336 | no |
| 130.05094- | 5-Amino-2-oxopentanoic acid | C01110 | [M-H]1- | 0.126504975581917 | no |
| 130.05094- | Hydroxyproline | C01157 | [M-H]1- | 0.126504975581917 | no |
| 130.05094- | 2-Amino-4-oxopentanoic acid | C03341 | [M-H]1- | 0.126504975581917 | no |
| 130.05094- | cis-4-Hydroxy-D-proline | C03440 | [M-H]1- | 0.126504975581917 | no |
| 129.01939- | 2,5-Dioxopentanoate | C00433 | [M-H]1- | 0.515055070222811 | no |
| 128.03539- | 5-Oxo-D-proline | C02237 | [M-H]1- | 0.519013461863755 | no |
| 128.03539- | 1-Pyrroline-4-hydroxy-2-carboxylate | C04282 | [M-H]1- | 0.519013461863755 | no |
| 128.03177 | L-Serine | C00065 | [M+Na]1+ | 0.39896418676284 | no |
| 128.03177 | D-Serine | C00740 | [M+Na]1+ | 0.39896418676284 | no |
| 120.06550 | L-Threonine | C00188 | [M+H]1+ | 0.196125523111715 | no |
| 120.06550 | D-Threonine | C00820 | [M+H]1+ | 0.196125523111715 | no |
| 120.01245- | L-Cysteine | C00097 | [M-H]1- | 0.220411224056257 | no |
| 120.01245- | D-Cysteine | C00793 | [M-H]1- | 0.220411224056257 | no |
| 118.05102- | L-Threonine | C00188 | [M-H]1- | 0.817037336909817 | no |
| 118.05102- | D-Threonine | C00820 | [M-H]1- | 0.817037336909817 | no |
| 116.07170- | 5-Aminopentanoate | C00431 | [M-H]1- | 0.202873818346407 | no |
| 116.07060 | L-Proline | C00148 | [M+H]1+ | 0.202875741102136 | no |
| 116.07060 | D-Proline | C00763 | [M+H]1+ | 0.202875741102136 | no |
| 114.05601- | L-Proline | C00148 | [M-H]1- | 0.118782947009942 | no |
| 114.05601- | D-Proline | C00763 | [M-H]1- | 0.118782947009942 | no |
| 112.04042- | 1-Pyrroline-2-carboxylate | C03564 | [M-H]1- | 0.0316663308030455 | no |
| 112.03925 | Pyrrole-2-carboxylate | C05942 | [M+H]1+ | 0.236096644760567 | no |
| 112.03688 | L-Alanine | C00041 | [M+Na]1+ | 0.366665789058869 | no |
| 112.03688 | D-Alanine | C00133 | [M+Na]1+ | 0.366665789058869 | no |
| 111.00527 | Pyruvate | C00022 | [M+Na]1+ | 0.440698977546308 | no |
| 110.02474- | Pyrrole-2-carboxylate | C05942 | [M-H]1- | 0.149530832822837 | no |
| 104.03534- | L-Serine | C00065 | [M-H]1- | 0.158139445860676 | no |
| 104.03534- | D-Serine | C00740 | [M-H]1- | 0.158139445860676 | no |

## Table S5 – *Valine, leucine and isoleucine degradation* hits

| **m/z** | **compoundname** | **identifier** | **adduct** | **Δppm** | **in sig** |
| --- | --- | --- | --- | --- | --- |
| 154.08382 | L-Leucine | C00123 | [M+Na]1+ | 0.00700976267597773 | yes |
| 132.10192 | L-Leucine | C00123 | [M+H]1+ | 0.329653868781906 | yes |
| 117.05461 | 3-Methyl-2-oxobutanoic acid | C00141 | [M+H]1+ | 0.286600493428946 | yes |
| 103.03895 | Acetoacetate | C00164 | [M+H]1+ | 0.256719337750339 | yes |
| 118.08619 | L-Valine | C00183 | [M+H]1+ | 0.732110088377933 | yes |
| 131.07031 | 4-Methyl-2-oxopentanoate | C00233 | [M+H]1+ | 0.255953533845807 | yes |
| 103.03895 | 2-Methyl-3-oxopropanoate | C00349 | [M+H]1+ | 0.256719337750339 | yes |
| 154.08382 | L-Isoleucine | C00407 | [M+Na]1+ | 0.00700976267597773 | yes |
| 132.10192 | L-Isoleucine | C00407 | [M+H]1+ | 0.329653868781906 | yes |
| 131.07031 | (S)-3-Methyl-2-oxopentanoic acid | C00671 | [M+H]1+ | 0.255953533845807 | yes |
| 104.07061 | (R)-3-Amino-2-methylpropanoate | C01205 | [M+H]1+ | 0.322357186129145 | yes |
| 176.99245 | Methylmalonate | C02170 | [M+NaCl+H]1+ | 0.275842788790572 | yes |
| 104.07061 | L-3-Aminoisobutanoate | C03284 | [M+H]1+ | 0.322357186129145 | yes |
| 127.03656 | (S)-3-Hydroxyisobutyrate | C06001 | [M+Na]1+ | 0.306367780960675 | yes |
| 103.03895 | (S)-Methylmalonate semialdehyde | C06002 | [M+H]1+ | 0.256719337750339 | yes |
| 119.07025 | 3-Hydroxyisovalerate | C20827 | [M+H]1+ | 0.222155332725767 | yes |
| 130.08732- | L-Leucine | C00123 | [M-H]1- | 0.0272732885668104 | no |
| 166.06408- | L-Leucine | C00123 | [M+Cl]1- | 0.473974209080673 | no |
| 188.04605- | L-Leucine | C00123 | [M+NaCl-H]1- | 0.553492567299356 | no |
| 139.03659 | 3-Methyl-2-oxobutanoic acid | C00141 | [M+Na]1+ | 0.49569619755051 | no |
| 115.04009- | 3-Methyl-2-oxobutanoic acid | C00141 | [M-H]1- | 0.577642898271168 | no |
| 125.02091 | Acetoacetate | C00164 | [M+Na]1+ | 0.0886259026539752 | no |
| 101.02439- | Acetoacetate | C00164 | [M-H]1- | 0.332077323093401 | no |
| 140.06821 | L-Valine | C00183 | [M+Na]1+ | 0.0791049661702838 | no |
| 116.07170- | L-Valine | C00183 | [M-H]1- | 0.202873818346407 | no |
| 174.03035- | L-Valine | C00183 | [M+NaCl-H]1- | 0.0234562018067808 | no |
| 152.04836- | L-Valine | C00183 | [M+Cl]1- | 0.271557739962053 | no |
| 153.05222 | 4-Methyl-2-oxopentanoate | C00233 | [M+Na]1+ | 0.00705700957481283 | no |
| 187.01440- | 4-Methyl-2-oxopentanoate | C00233 | [M+NaCl-H]1- | 0.289186773839628 | no |
| 129.05582- | 4-Methyl-2-oxopentanoate | C00233 | [M-H]1- | 0.747367232368896 | no |
| 165.03245- | 4-Methyl-2-oxopentanoate | C00233 | [M+Cl]1- | 0.295154625784387 | no |
| 125.02091 | 2-Methyl-3-oxopropanoate | C00349 | [M+Na]1+ | 0.0886259026539752 | no |
| 101.02439- | 2-Methyl-3-oxopropanoate | C00349 | [M-H]1- | 0.332077323093401 | no |
| 130.08732- | L-Isoleucine | C00407 | [M-H]1- | 0.0272732885668104 | no |
| 166.06408- | L-Isoleucine | C00407 | [M+Cl]1- | 0.473974209080673 | no |
| 188.04605- | L-Isoleucine | C00407 | [M+NaCl-H]1- | 0.553492567299356 | no |
| 153.05222 | (S)-3-Methyl-2-oxopentanoic acid | C00671 | [M+Na]1+ | 0.00705700957481283 | no |
| 187.01440- | (S)-3-Methyl-2-oxopentanoic acid | C00671 | [M+NaCl-H]1- | 0.289186773839628 | no |
| 129.05582- | (S)-3-Methyl-2-oxopentanoic acid | C00671 | [M-H]1- | 0.747367232368896 | no |
| 165.03245- | (S)-3-Methyl-2-oxopentanoic acid | C00671 | [M+Cl]1- | 0.295154625784387 | no |
| 126.05254 | (R)-3-Amino-2-methylpropanoate | C01205 | [M+Na]1+ | 0.150095420496407 | no |
| 102.05604- | (R)-3-Amino-2-methylpropanoate | C01205 | [M-H]1- | 0.161206441121134 | no |
| 141.01591 | Methylmalonate | C02170 | [M+Na]1+ | 0.63056650125321 | no |
| 117.01939- | Methylmalonate | C02170 | [M-H]1- | 0.567872478270276 | no |
| 174.97798- | Methylmalonate | C02170 | [M+NaCl-H]1- | 0.194779314466914 | no |
| 152.99593- | Methylmalonate | C02170 | [M+Cl]1- | 0.46595951288416 | no |
| 126.05254 | L-3-Aminoisobutanoate | C03284 | [M+Na]1+ | 0.150095420496407 | no |
| 102.05604- | L-3-Aminoisobutanoate | C03284 | [M-H]1- | 0.161206441121134 | no |
| 105.05468 | (S)-3-Hydroxyisobutyrate | C06001 | [M+H]1+ | 0.985657269073029 | no |
| 160.99873- | (S)-3-Hydroxyisobutyrate | C06001 | [M+NaCl-H]1- | 0.522253131926285 | no |
| 103.04011- | (S)-3-Hydroxyisobutyrate | C06001 | [M-H]1- | 0.839013962592949 | no |
| 139.01672- | (S)-3-Hydroxyisobutyrate | C06001 | [M+Cl]1- | 0.134588781724144 | no |
| 125.02091 | (S)-Methylmalonate semialdehyde | C06002 | [M+Na]1+ | 0.0886259026539752 | no |
| 101.02439- | (S)-Methylmalonate semialdehyde | C06002 | [M-H]1- | 0.332077323093401 | no |
| 141.05220 | 3-Hydroxyisovalerate | C20827 | [M+Na]1+ | 0.149448863529492 | no |
| 153.03241- | 3-Hydroxyisovalerate | C20827 | [M+Cl]1- | 0.0569166427512581 | no |
| 175.01434- | 3-Hydroxyisovalerate | C20827 | [M+NaCl-H]1- | 0.0338138520138946 | no |
| 117.05575- | 3-Hydroxyisovalerate | C20827 | [M-H]1- | 0.22597857009579 | no |

## Table S6 – *Glycine, serine and threonine metabolism* hits

| **m/z** | **compoundname** | **identifier** | **adduct** | **Δppm** | **in sig** |
| --- | --- | --- | --- | --- | --- |
| 134.04478 | L-Aspartate | C00049 | [M+H]1+ | 0.0264680878825942 | yes |
| 156.02673 | L-Aspartate | C00049 | [M+Na]1+ | 0.057169108092992 | yes |
| 164.00832 | L-Serine | C00065 | [M+NaCl+H]1+ | 1.09032328961906 | yes |
| 106.04983 | L-Serine | C00065 | [M+H]1+ | 0.438021362208951 | yes |
| 205.09718 | L-Tryptophan | C00078 | [M+H]1+ | 0.01729867284008 | yes |
| 122.02695 | L-Cysteine | C00097 | [M+H]1+ | 0.216772532678153 | yes |
| 103.03895 | 2-Oxobutanoate | C00109 | [M+H]1+ | 0.256719337750339 | yes |
| 142.04751 | L-Threonine | C00188 | [M+Na]1+ | 0.625987101004006 | yes |
| 208.98225 | 3-Phospho-D-glycerate | C00197 | [M+Na]1+ | 0.616894061588647 | yes |
| 187.00054 | 3-Phospho-D-glycerate | C00197 | [M+H]1+ | 1.94410085135489 | yes |
| 164.99247 | D-Glycerate | C00258 | [M+NaCl+H]1+ | 0.174687311466563 | yes |
| 142.04751 | L-Homoserine | C00263 | [M+Na]1+ | 0.625987101004006 | yes |
| 154.05868 | Creatine | C00300 | [M+Na]1+ | 0.26665223273809 | yes |
| 132.07679 | Creatine | C00300 | [M+H]1+ | 0.102576001370077 | yes |
| 132.06558 | 5-Aminolevulinate | C00430 | [M+H]1+ | 0.784064318807088 | yes |
| 154.04745 | 5-Aminolevulinate | C00430 | [M+Na]1+ | 0.187733772932394 | yes |
| 176.00844 | L-Aspartate 4-semialdehyde | C00441 | [M+NaCl+H]1+ | 0.334200399580999 | yes |
| 73.02844 | Methylglyoxal | C00546 | [M+H]1+ | 0.870180288578741 | yes |
| 208.98225 | 2-Phospho-D-glycerate | C00631 | [M+Na]1+ | 0.616894061588647 | yes |
| 187.00054 | 2-Phospho-D-glycerate | C00631 | [M+H]1+ | 1.94410085135489 | yes |
| 118.08619 | Betaine | C00719 | [M+H]1+ | 0.732110088377933 | yes |
| 164.00832 | D-Serine | C00740 | [M+NaCl+H]1+ | 1.09032328961906 | yes |
| 106.04983 | D-Serine | C00740 | [M+H]1+ | 0.438021362208951 | yes |
| 75.09169 | 1,3-Diaminopropane | C00986 | [M+H]1+ | 0.180418219272937 | yes |
| 104.07061 | N,N-Dimethylglycine | C01026 | [M+H]1+ | 0.322357186129145 | yes |
| 74.06007 | Aminoacetone | C01888 | [M+H]1+ | 0.0871196991084784 | yes |
| 76.07569 | (R)-1-Aminopropan-2-ol | C03194 | [M+H]1+ | 0.178084602216495 | yes |
| 176.00844 | L-2-Amino-3-oxobutanoic acid | C03508 | [M+NaCl+H]1+ | 0.334200399580999 | yes |
| 142.04751 | L-Allothreonine | C05519 | [M+Na]1+ | 0.625987101004006 | yes |
| 165.06343 | Ectoine | C06231 | [M+Na]1+ | 0.0540392806643348 | yes |
| 143.08154 | Ectoine | C06231 | [M+H]1+ | 0.444137720406602 | yes |
| 161.09202 | N(gamma)-Acetyldiaminobutyrate | C06442 | [M+H]1+ | 0.350433814175288 | yes |
| 183.07413 | N(gamma)-Acetyldiaminobutyrate | C06442 | [M+Na]1+ | 0.59494975612385 | yes |
| 161.09202 | N(alpha)-Acetyl-L-2,4-diaminobutyrate | C19929 | [M+H]1+ | 0.350433814175288 | yes |
| 183.07413 | N(alpha)-Acetyl-L-2,4-diaminobutyrate | C19929 | [M+Na]1+ | 0.59494975612385 | yes |
| 78.95908- | CO2 | C00011 | [M+Cl]1- | 1.53611097043567 | no |
| 89.02327 | Pyruvate | C00022 | [M+H]1+ | 0.0724764549128879 | no |
| 111.00527 | Pyruvate | C00022 | [M+Na]1+ | 0.440698977546308 | no |
| 87.00878- | Pyruvate | C00022 | [M-H]1- | 0.648809131696845 | no |
| 144.96734- | Pyruvate | C00022 | [M+NaCl-H]1- | 0.0408223603541972 | no |
| 76.03930 | Glycine | C00037 | [M+H]1+ | 0.309680770274971 | no |
| 98.02124 | Glycine | C00037 | [M+Na]1+ | 0.193018462224627 | no |
| 131.98335- | Glycine | C00037 | [M+NaCl-H]1- | 0.030928833145277 | no |
| 74.02475- | Glycine | C00037 | [M-H]1- | 0.357341173141285 | no |
| 72.99309- | Glyoxylate | C00048 | [M-H]1- | 0.459603902209698 | no |
| 130.95178- | Glyoxylate | C00048 | [M+NaCl-H]1- | 0.260264434890668 | no |
| 192.00331 | L-Aspartate | C00049 | [M+NaCl+H]1+ | 0.462607082071401 | no |
| 168.00693- | L-Aspartate | C00049 | [M+Cl]1- | 0.17088634984094 | no |
| 189.98896- | L-Aspartate | C00049 | [M+NaCl-H]1- | 0.600466948122549 | no |
| 132.03027- | L-Aspartate | C00049 | [M-H]1- | 0.35182909948323 | no |
| 128.03177 | L-Serine | C00065 | [M+Na]1+ | 0.39896418676284 | no |
| 140.01190- | L-Serine | C00065 | [M+Cl]1- | 0.723437857761805 | no |
| 104.03534- | L-Serine | C00065 | [M-H]1- | 0.158139445860676 | no |
| 161.99399- | L-Serine | C00065 | [M+NaCl-H]1- | 0.272121768186459 | no |
| 227.07910 | L-Tryptophan | C00078 | [M+Na]1+ | 0.0928314890640953 | no |
| 263.05613 | L-Tryptophan | C00078 | [M+NaCl+H]1+ | 1.25896290272367 | no |
| 203.08272- | L-Tryptophan | C00078 | [M-H]1- | 0.474939920964604 | no |
| 239.05968- | L-Tryptophan | C00078 | [M+Cl]1- | 1.58416547275839 | no |
| 261.04142- | L-Tryptophan | C00078 | [M+NaCl-H]1- | 0.666875360267432 | no |
| 120.01245- | L-Cysteine | C00097 | [M-H]1- | 0.220411224056257 | no |
| 125.02091 | 2-Oxobutanoate | C00109 | [M+Na]1+ | 0.0886259026539752 | no |
| 101.02439- | 2-Oxobutanoate | C00109 | [M-H]1- | 0.332077323093401 | no |
| 127.00040 | Hydroxypyruvate | C00168 | [M+Na]1+ | 1.40881374392596 | no |
| 103.00368- | Hydroxypyruvate | C00168 | [M-H]1- | 0.422780127732398 | no |
| 120.06550 | L-Threonine | C00188 | [M+H]1+ | 0.196125523111715 | no |
| 154.02766- | L-Threonine | C00188 | [M+Cl]1- | 0.381165895766923 | no |
| 118.05102- | L-Threonine | C00188 | [M-H]1- | 0.817037336909817 | no |
| 176.00963- | L-Threonine | C00188 | [M+NaCl-H]1- | 0.477713014785921 | no |
| 184.98573- | 3-Phospho-D-glycerate | C00197 | [M-H]1- | 0.575461096319435 | no |
| 90.05496 | Sarcosine | C00213 | [M+H]1+ | 0.182689448949031 | no |
| 112.03688 | Sarcosine | C00213 | [M+Na]1+ | 0.366665789058869 | no |
| 88.04044- | Sarcosine | C00213 | [M-H]1- | 0.186869704459861 | no |
| 145.99898- | Sarcosine | C00213 | [M+NaCl-H]1- | 0.451495681765135 | no |
| 140.99602- | D-Glycerate | C00258 | [M+Cl]1- | 0.132699426438324 | no |
| 105.01930- | D-Glycerate | C00258 | [M-H]1- | 0.224224585339739 | no |
| 162.97803- | D-Glycerate | C00258 | [M+NaCl-H]1- | 0.515910586099577 | no |
| 120.06550 | L-Homoserine | C00263 | [M+H]1+ | 0.196125523111715 | no |
| 154.02766- | L-Homoserine | C00263 | [M+Cl]1- | 0.381165895766923 | no |
| 118.05102- | L-Homoserine | C00263 | [M-H]1- | 0.817037336909817 | no |
| 176.00963- | L-Homoserine | C00263 | [M+NaCl-H]1- | 0.477713014785921 | no |
| 130.06215- | Creatine | C00300 | [M-H]1- | 0.565482801960757 | no |
| 188.02084- | Creatine | C00300 | [M+NaCl-H]1- | 0.0314747504664437 | no |
| 166.03890- | Creatine | C00300 | [M+Cl]1- | 0.00776871560724667 | no |
| 166.02767- | 5-Aminolevulinate | C00430 | [M+Cl]1- | 0.413847227994931 | no |
| 130.05094- | 5-Aminolevulinate | C00430 | [M-H]1- | 0.126504975581917 | no |
| 140.03187 | L-Aspartate 4-semialdehyde | C00441 | [M+Na]1+ | 0.349348394800859 | no |
| 116.03533- | L-Aspartate 4-semialdehyde | C00441 | [M-H]1- | 0.0556045388447885 | no |
| 71.01378- | Methylglyoxal | C00546 | [M-H]1- | 0.613231812213317 | no |
| 184.98573- | 2-Phospho-D-glycerate | C00631 | [M-H]1- | 0.575461096319435 | no |
| 140.06821 | Betaine | C00719 | [M+Na]1+ | 0.0791049661702838 | no |
| 116.07170- | Betaine | C00719 | [M-H]1- | 0.202873818346407 | no |
| 174.03035- | Betaine | C00719 | [M+NaCl-H]1- | 0.0234562018067808 | no |
| 152.04836- | Betaine | C00719 | [M+Cl]1- | 0.271557739962053 | no |
| 128.03177 | D-Serine | C00740 | [M+Na]1+ | 0.39896418676284 | no |
| 140.01190- | D-Serine | C00740 | [M+Cl]1- | 0.723437857761805 | no |
| 104.03534- | D-Serine | C00740 | [M-H]1- | 0.158139445860676 | no |
| 161.99399- | D-Serine | C00740 | [M+NaCl-H]1- | 0.272121768186459 | no |
| 207.99826 | O-Phospho-L-serine | C01005 | [M+Na]1+ | 0.667889764929697 | no |
| 186.01636 | O-Phospho-L-serine | C01005 | [M+H]1+ | 0.986729925201805 | no |
| 241.96057- | O-Phospho-L-serine | C01005 | [M+NaCl-H]1- | 1.33940042780346 | no |
| 219.97844- | O-Phospho-L-serine | C01005 | [M+Cl]1- | 0.630562208704426 | no |
| 126.05254 | N,N-Dimethylglycine | C01026 | [M+Na]1+ | 0.150095420496407 | no |
| 102.05604- | N,N-Dimethylglycine | C01026 | [M-H]1- | 0.161206441121134 | no |
| 200.03199 | O-Phospho-L-homoserine | C01102 | [M+H]1+ | 0.56764874970117 | no |
| 198.01750- | O-Phospho-L-homoserine | C01102 | [M-H]1- | 0.891093418568914 | no |
| 96.04193 | Aminoacetone | C01888 | [M+Na]1+ | 0.94833674219743 | no |
| 98.05760 | (R)-1-Aminopropan-2-ol | C03194 | [M+Na]1+ | 0.214976615810967 | no |
| 240.92880- | 3-Phosphonooxypyruvate | C03232 | [M+NaCl-H]1- | 0.639533716900811 | no |
| 140.03187 | L-2-Amino-3-oxobutanoic acid | C03508 | [M+Na]1+ | 0.349348394800859 | no |
| 116.03533- | L-2-Amino-3-oxobutanoic acid | C03508 | [M-H]1- | 0.0556045388447885 | no |
| 120.06550 | L-Allothreonine | C05519 | [M+H]1+ | 0.196125523111715 | no |
| 154.02766- | L-Allothreonine | C05519 | [M+Cl]1- | 0.381165895766923 | no |
| 118.05102- | L-Allothreonine | C05519 | [M-H]1- | 0.817037336909817 | no |
| 176.00963- | L-Allothreonine | C05519 | [M+NaCl-H]1- | 0.477713014785921 | no |
| 141.06697- | Ectoine | C06231 | [M-H]1- | 0.329291052318113 | no |
| 195.05406- | N(gamma)-Acetyldiaminobutyrate | C06442 | [M+Cl]1- | 0.724362820286109 | no |
| 159.07751- | N(gamma)-Acetyldiaminobutyrate | C06442 | [M-H]1- | 0.0851654581494084 | no |
| 181.05822 | 5-Hydroxyectoine | C16432 | [M+Na]1+ | 0.558273968369434 | no |
| 159.07640 | 5-Hydroxyectoine | C16432 | [M+H]1+ | 0.148028928386557 | no |
| 157.06185- | 5-Hydroxyectoine | C16432 | [M-H]1- | 0.168418307770343 | no |
| 195.05406- | N(alpha)-Acetyl-L-2,4-diaminobutyrate | C19929 | [M+Cl]1- | 0.724362820286109 | no |
| 159.07751- | N(alpha)-Acetyl-L-2,4-diaminobutyrate | C19929 | [M-H]1- | 0.0851654581494084 | no |
|  |  |  |  |  |  |

## Table S7 – *Phenylalanine metabolism* hits

| **m/z** | **compoundname** | **identifier** | **adduct** | **Δppm** | **in sig** |
| --- | --- | --- | --- | --- | --- |
| 176.99245 | Succinate | C00042 | [M+NaCl+H]1+ | 0.275842788790572 | yes |
| 188.06823 | L-Phenylalanine | C00079 | [M+Na]1+ | 0.0474291111941984 | yes |
| 182.08124 | L-Tyrosine | C00082 | [M+H]1+ | 0.349008546997297 | yes |
| 204.06311 | L-Tyrosine | C00082 | [M+Na]1+ | 0.054297374050519 | yes |
| 165.05463 | Phenylpyruvate | C00166 | [M+H]1+ | 0.324425367556742 | yes |
| 149.05974 | trans-Cinnamate | C00423 | [M+H]1+ | 0.426325102994719 | yes |
| 171.04176 | trans-Cinnamate | C00423 | [M+Na]1+ | 0.812198781195937 | yes |
| 115.03892 | 2-Hydroxy-2,4-pentadienoate | C00596 | [M+H]1+ | 0.490721670559857 | yes |
| 121.06475 | Phenylacetaldehyde | C00601 | [M+H]1+ | 0.218495400242352 | yes |
| 153.05463 | 4-Hydroxyphenylacetate | C00642 | [M+H]1+ | 0.34986141225974 | yes |
| 175.03666 | 4-Hydroxyphenylacetate | C00642 | [M+Na]1+ | 0.793661790653501 | yes |
| 167.07027 | 3-(2-Hydroxyphenyl)propanoate | C01198 | [M+H]1+ | 0.038619025311512 | yes |
| 165.05463 | trans-2-Hydroxycinnamate | C01772 | [M+H]1+ | 0.324425367556742 | yes |
| 173.02111 | alpha-Oxo-benzeneacetic acid | C02137 | [M+Na]1+ | 1.09188935958772 | yes |
| 188.06823 | D-Phenylalanine | C02265 | [M+Na]1+ | 0.0474291111941984 | yes |
| 136.07569 | 2-Phenylacetamide | C02505 | [M+H]1+ | 0.0995615676864626 | yes |
| 165.05463 | 2-Hydroxy-3-phenylpropenoate | C02763 | [M+H]1+ | 0.324425367556742 | yes |
| 155.03146 | 4-Hydroxy-2-oxopentanoate | C03589 | [M+Na]1+ | 0.393985136958855 | yes |
| 191.00804 | 4-Hydroxy-2-oxopentanoate | C03589 | [M+NaCl+H]1+ | 0.83149427118026 | yes |
| 122.09639 | Phenethylamine | C05332 | [M+H]1+ | 0.110960766260128 | yes |
| 153.05463 | 3-Hydroxyphenylacetate | C05593 | [M+H]1+ | 0.34986141225974 | yes |
| 175.03666 | 3-Hydroxyphenylacetate | C05593 | [M+Na]1+ | 0.793661790653501 | yes |
| 167.07027 | Phenyllactate | C05607 | [M+H]1+ | 0.038619025311512 | yes |
| 153.05463 | 2-Hydroxyphenylacetate | C05852 | [M+H]1+ | 0.34986141225974 | yes |
| 175.03666 | 2-Hydroxyphenylacetate | C05852 | [M+Na]1+ | 0.793661790653501 | yes |
| 123.08039 | Phenylethyl alcohol | C05853 | [M+H]1+ | 0.702403453639725 | yes |
| 167.07027 | 3-(3-Hydroxyphenyl)propanoic acid | C11457 | [M+H]1+ | 0.038619025311512 | yes |
| 165.05463 | trans-3-Hydroxycinnamate | C12621 | [M+H]1+ | 0.324425367556742 | yes |
| 89.02327 | Pyruvate | C00022 | [M+H]1+ | 0.0724764549128879 | no |
| 111.00527 | Pyruvate | C00022 | [M+Na]1+ | 0.440698977546308 | no |
| 87.00878- | Pyruvate | C00022 | [M-H]1- | 0.648809131696845 | no |
| 144.96734- | Pyruvate | C00022 | [M+NaCl-H]1- | 0.0408223603541972 | no |
| 141.01591 | Succinate | C00042 | [M+Na]1+ | 0.63056650125321 | no |
| 117.01939- | Succinate | C00042 | [M-H]1- | 0.567872478270276 | no |
| 174.97798- | Succinate | C00042 | [M+NaCl-H]1- | 0.194779314466914 | no |
| 152.99593- | Succinate | C00042 | [M+Cl]1- | 0.46595951288416 | no |
| 166.08628 | L-Phenylalanine | C00079 | [M+H]1+ | 0.0213618427050163 | no |
| 200.04847- | L-Phenylalanine | C00079 | [M+Cl]1- | 0.343467215720056 | no |
| 222.03037- | L-Phenylalanine | C00079 | [M+NaCl-H]1- | 0.108463049392888 | no |
| 164.07162- | L-Phenylalanine | C00079 | [M-H]1- | 0.631114077014434 | no |
| 180.06669- | L-Tyrosine | C00082 | [M-H]1- | 0.369041553307578 | no |
| 238.02538- | L-Tyrosine | C00082 | [M+NaCl-H]1- | 0.563310059601732 | no |
| 216.04320- | L-Tyrosine | C00082 | [M+Cl]1- | 0.468840995676605 | no |
| 115.00367- | Fumarate | C00122 | [M-H]1- | 0.465619131897974 | no |
| 172.96231- | Fumarate | C00122 | [M+NaCl-H]1- | 0.207663212884887 | no |
| 150.98031- | Fumarate | C00122 | [M+Cl]1- | 0.604647778203566 | no |
| 187.03669 | Phenylpyruvate | C00166 | [M+Na]1+ | 0.903137822780701 | no |
| 163.04016- | Phenylpyruvate | C00166 | [M-H]1- | 0.83692319101227 | no |
| 147.04513- | trans-Cinnamate | C00423 | [M-H]1- | 0.0438783044765314 | no |
| 137.02099 | 2-Hydroxy-2,4-pentadienoate | C00596 | [M+Na]1+ | 0.502987965652641 | no |
| 113.02444- | 2-Hydroxy-2,4-pentadienoate | C00596 | [M-H]1- | 0.145562243027403 | no |
| 143.04683 | Phenylacetaldehyde | C00601 | [M+Na]1+ | 0.761428330820655 | no |
| 119.05018- | Phenylacetaldehyde | C00601 | [M-H]1- | 0.365794566640032 | no |
| 151.04004- | 4-Hydroxyphenylacetate | C00642 | [M-H]1- | 0.10892536170611 | no |
| 189.05235 | 3-(2-Hydroxyphenyl)propanoate | C01198 | [M+Na]1+ | 0.6819270376826 | no |
| 165.05572- | 3-(2-Hydroxyphenyl)propanoate | C01198 | [M-H]1- | 0.0214952198495229 | no |
| 187.03669 | trans-2-Hydroxycinnamate | C01772 | [M+Na]1+ | 0.903137822780701 | no |
| 163.04016- | trans-2-Hydroxycinnamate | C01772 | [M-H]1- | 0.83692319101227 | no |
| 149.02435- | alpha-Oxo-benzeneacetic acid | C02137 | [M-H]1- | 0.493529473613139 | no |
| 166.08628 | D-Phenylalanine | C02265 | [M+H]1+ | 0.0213618427050163 | no |
| 200.04847- | D-Phenylalanine | C02265 | [M+Cl]1- | 0.343467215720056 | no |
| 222.03037- | D-Phenylalanine | C02265 | [M+NaCl-H]1- | 0.108463049392888 | no |
| 164.07162- | D-Phenylalanine | C02265 | [M-H]1- | 0.631114077014434 | no |
| 158.05766 | 2-Phenylacetamide | C02505 | [M+Na]1+ | 0.246238676456987 | no |
| 134.06117- | 2-Phenylacetamide | C02505 | [M-H]1- | 0.346499221203483 | no |
| 187.03669 | 2-Hydroxy-3-phenylpropenoate | C02763 | [M+Na]1+ | 0.903137822780701 | no |
| 163.04016- | 2-Hydroxy-3-phenylpropenoate | C02763 | [M-H]1- | 0.83692319101227 | no |
| 230.07863 | N-Acetyl-L-phenylalanine | C03519 | [M+Na]1+ | 0.395865061358075 | no |
| 206.08238- | N-Acetyl-L-phenylalanine | C03519 | [M-H]1- | 0.759172574604006 | no |
| 133.04954 | 4-Hydroxy-2-oxopentanoate | C03589 | [M+H]1+ | 0.273973822047438 | no |
| 131.03497- | 4-Hydroxy-2-oxopentanoate | C03589 | [M-H]1- | 0.408653575706651 | no |
| 188.99396- | 4-Hydroxy-2-oxopentanoate | C03589 | [M+NaCl-H]1- | 1.66186311438944 | no |
| 205.04726 | 3-(2,3-Dihydroxyphenyl)propanoate | C04044 | [M+Na]1+ | 0.677501903734817 | no |
| 181.05074- | 3-(2,3-Dihydroxyphenyl)propanoate | C04044 | [M-H]1- | 0.643201408579855 | no |
| 213.04059- | 2-Hydroxy-6-oxonona-2,4-diene-1,9-dioate | C04479 | [M-H]1- | 0.781316325721291 | no |
| 151.04004- | 3-Hydroxyphenylacetate | C05593 | [M-H]1- | 0.10892536170611 | no |
| 194.08126 | Phenylacetylglycine | C05598 | [M+H]1+ | 0.430479011656214 | no |
| 216.06313 | Phenylacetylglycine | C05598 | [M+Na]1+ | 0.0412838090088118 | no |
| 228.04357- | Phenylacetylglycine | C05598 | [M+Cl]1- | 1.17832785628718 | no |
| 192.06671- | Phenylacetylglycine | C05598 | [M-H]1- | 0.450114915712892 | no |
| 189.05235 | Phenyllactate | C05607 | [M+Na]1+ | 0.6819270376826 | no |
| 165.05572- | Phenyllactate | C05607 | [M-H]1- | 0.0214952198495229 | no |
| 173.05750 | Phenylpropanoate | C05629 | [M+Na]1+ | 1.03387549813389 | no |
| 151.07538 | Phenylpropanoate | C05629 | [M+H]1+ | 0.0234843626886327 | no |
| 149.06077- | Phenylpropanoate | C05629 | [M-H]1- | 0.359235424617034 | no |
| 151.04004- | 2-Hydroxyphenylacetate | C05852 | [M-H]1- | 0.10892536170611 | no |
| 145.06240 | Phenylethyl alcohol | C05853 | [M+Na]1+ | 0.145317401258594 | no |
| 169.04952 | 2,6-Dihydroxyphenylacetate | C06207 | [M+H]1+ | 0.33393819161274 | no |
| 167.03494- | 2,6-Dihydroxyphenylacetate | C06207 | [M-H]1- | 0.500182231419453 | no |
| 137.05973 | Phenylacetic acid | C07086 | [M+H]1+ | 0.390690314395716 | no |
| 159.04176 | Phenylacetic acid | C07086 | [M+Na]1+ | 0.873480707240715 | no |
| 135.04526- | Phenylacetic acid | C07086 | [M-H]1- | 1.01041747780539 | no |
| 189.05235 | 3-(3-Hydroxyphenyl)propanoic acid | C11457 | [M+Na]1+ | 0.6819270376826 | no |
| 165.05572- | 3-(3-Hydroxyphenyl)propanoic acid | C11457 | [M-H]1- | 0.0214952198495229 | no |
| 185.08085 | cis-3-(Carboxy-ethyl)-3,5-cyclo-hexadiene-1,2-diol | C11588 | [M+H]1+ | 0.142921814938063 | no |
| 207.06282 | cis-3-(Carboxy-ethyl)-3,5-cyclo-hexadiene-1,2-diol | C11588 | [M+Na]1+ | 0.00521624795029871 | no |
| 183.06630- | cis-3-(Carboxy-ethyl)-3,5-cyclo-hexadiene-1,2-diol | C11588 | [M-H]1- | 0.128630496138118 | no |
| 187.03669 | trans-3-Hydroxycinnamate | C12621 | [M+Na]1+ | 0.903137822780701 | no |
| 163.04016- | trans-3-Hydroxycinnamate | C12621 | [M-H]1- | 0.83692319101227 | no |
| 205.04726 | cis-3-(3-Carboxyethenyl)-3,5-cyclohexadiene-1,2-diol | C12622 | [M+Na]1+ | 0.677501903734817 | no |
| 181.05074- | cis-3-(3-Carboxyethenyl)-3,5-cyclohexadiene-1,2-diol | C12622 | [M-H]1- | 0.643201408579855 | no |
| 203.03167 | trans-2,3-Dihydroxycinnamate | C12623 | [M+Na]1+ | 0.733481180450129 | no |
| 179.03510- | trans-2,3-Dihydroxycinnamate | C12623 | [M-H]1- | 0.427022918856294 | no |
| 235.02142 | 2-Hydroxy-6-ketononatrienedioate | C12624 | [M+Na]1+ | 0.420897418596618 | no |


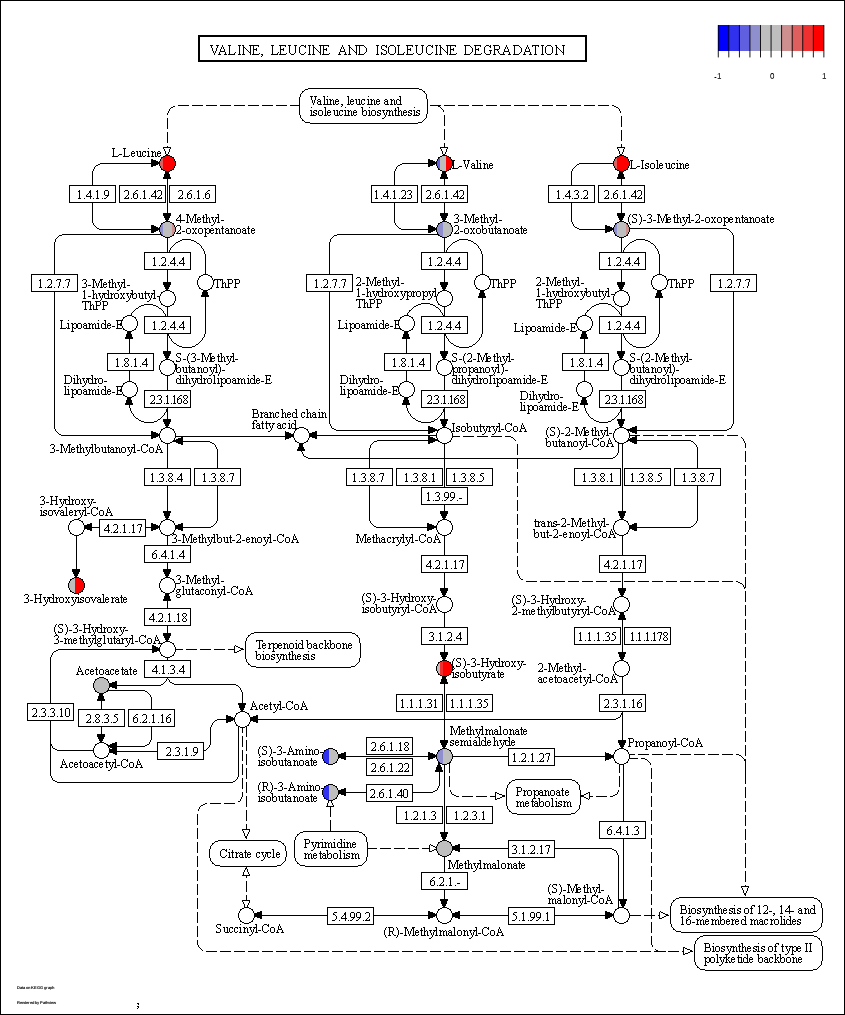


## Figure S2

Color of the node represents its V-score . Grayed out nodes are present in data but not in signature. Red represents V-score above 0 (higher in unhealthy chickens) and blue represents V-score below 0 (higher in high health chickens). Generated in R using the *pathview* package.


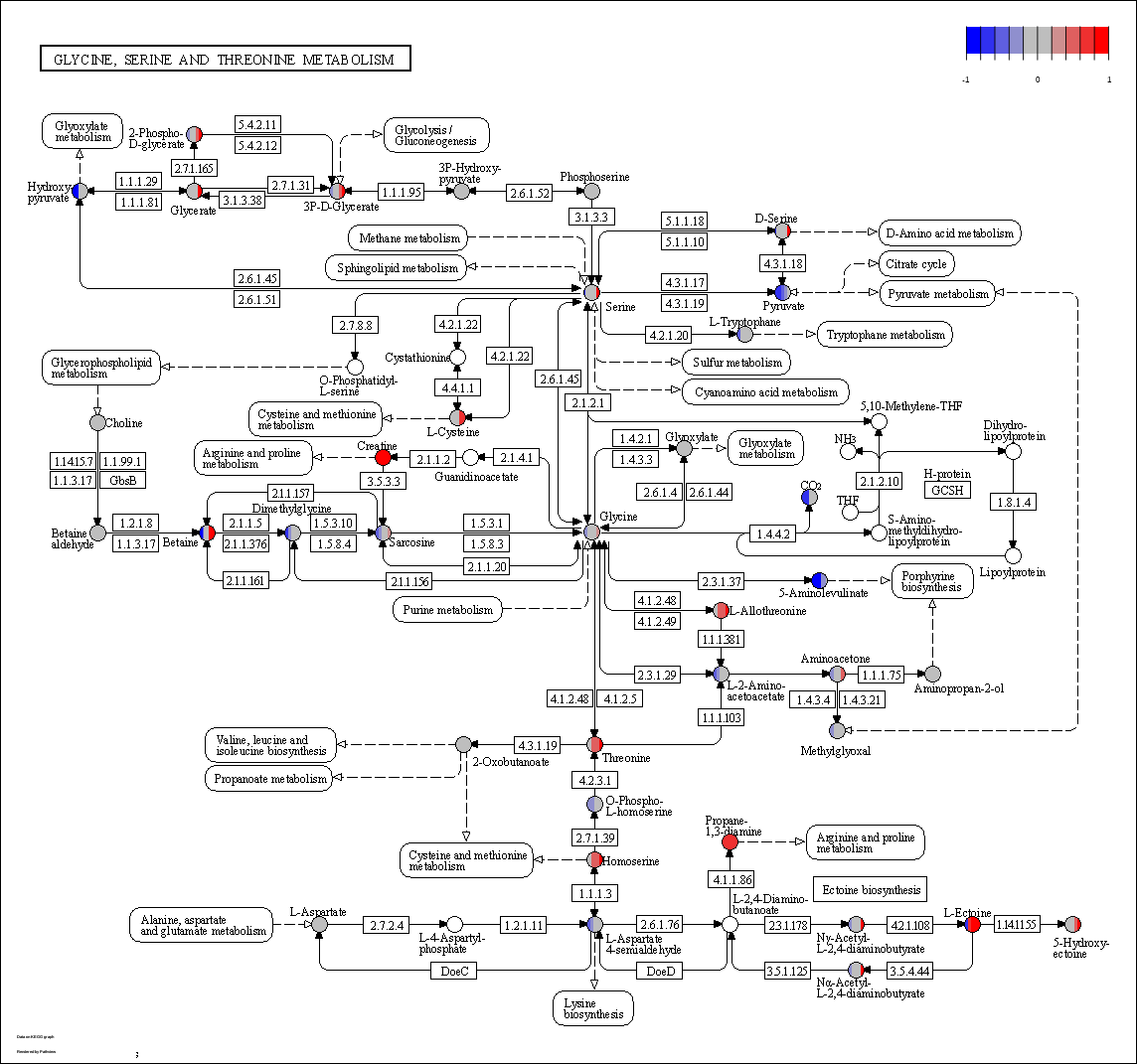


## Figure S3

Color of the node represents its V-score . Grayed out nodes are present in data but not in signature. Red represents V-score above 0 (higher in unhealthy chickens) and blue represents V-score below 0 (higher in high health chickens). Generated in R using the *pathview* package.


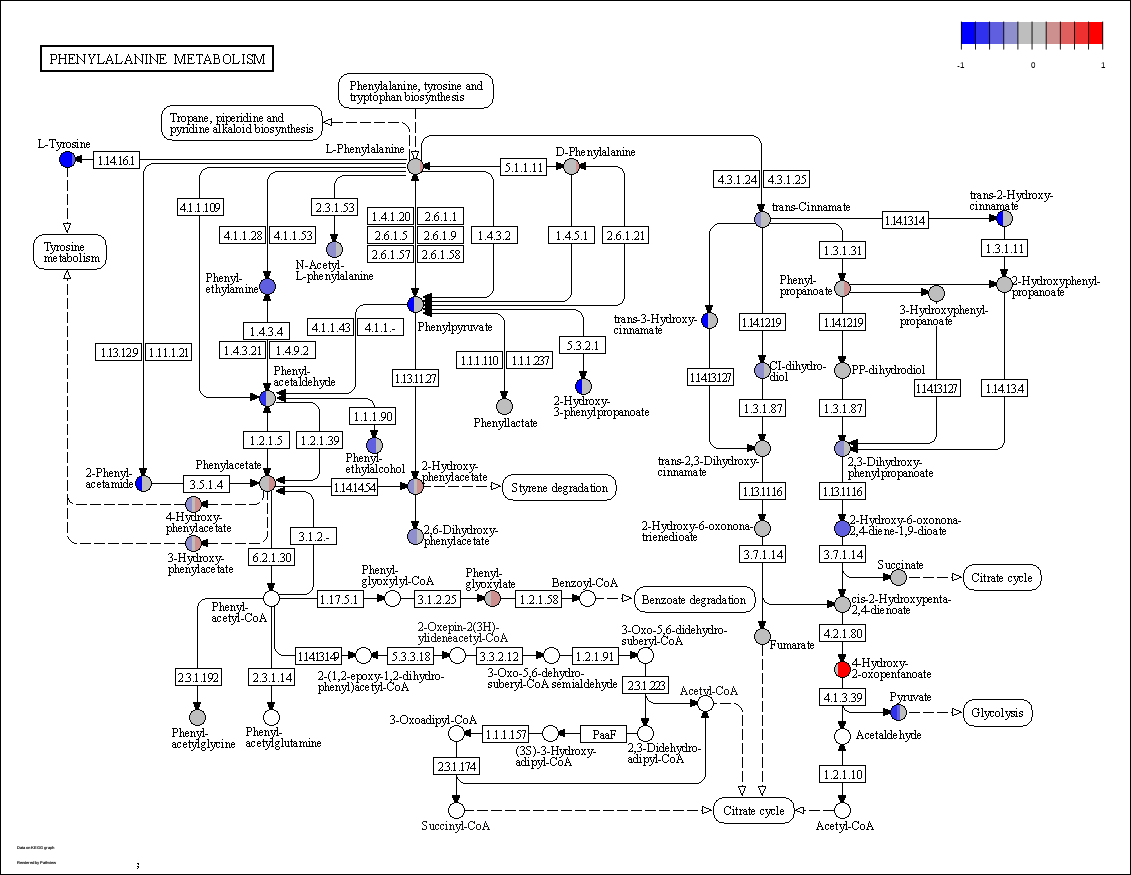


## Figure S4

Color of the node represents its V-score . Grayed out nodes are present in data but not in signature. Red represents V-score above 0 (higher in unhealthy chickens) and blue represents V-score below 0 (higher in high health chickens). Generated in R using the *pathview* package.
